# Gut Non-Bacterial Microbiota: Emerging Link to Irritable Bowel Syndrome

**DOI:** 10.3390/toxins14090596

**Published:** 2022-08-29

**Authors:** Ao Liu, Wenkang Gao, Yixin Zhu, Xiaohua Hou, Huikuan Chu

**Affiliations:** 1 Division of Gastroenterology, Union Hospital, Tongji Medical College, Huazhong University of Science and Technology, 1277 Jiefang Avenue, Wuhan 430022, China; 2 Department of Medicine, University of California San Diego, La Jolla, CA 92093, USA

**Keywords:** irritable bowel syndrome, microbiome, metabolites, diagnostic tests

## Abstract

As a common functional gastrointestinal disorder, irritable bowel syndrome (IBS) significantly affects personal health and imposes a substantial economic burden on society, but the current understanding of its occurrence and treatment is still inadequate. Emerging evidence suggests that IBS is associated with gut microbial dysbiosis, but most studies focus on the bacteria and neglect other communities of the microbiota, including fungi, viruses, archaea, and other parasitic microorganisms. This review summarizes the latest findings that link the nonbacterial microbiota with IBS. IBS patients show less fungal and viral diversity but some alterations in mycobiome, virome, and archaeome, such as an increased abundance of *Candida albicans*. Moreover, fungi and methanogens can aid in diagnosis. Fungi are related to distinct IBS symptoms and induce immune responses, intestinal barrier disruption, and visceral hypersensitivity via specific receptors, cells, and metabolites. Novel therapeutic methods for IBS include fungicides, inhibitors targeting fungal pathogenic pathways, probiotic fungi, prebiotics, and fecal microbiota transplantation. Additionally, viruses, methanogens, and parasitic microorganisms are also involved in the pathophysiology and treatment. Therefore, the gut nonbacterial microbiota is involved in the pathogenesis of IBS, which provides a novel perspective on the noninvasive diagnosis and precise treatment of this disease.

## 1. Introduction

Irritable bowel syndrome (IBS) is a common functional gastrointestinal disorder characterized by recurrent abdominal pain related to defecation or altered bowel habits [1]. The prevalence rates of IBS on different continents range from 5.8% to 17.5% [2], and IBS significantly affects patients’ quality of life and increases healthcare costs. Unfortunately, the etiology of the disease remains unclear, and there is still great need of an effective clinical treatment method. Sometimes gastrointestinal infections, psychological factors, and bad living habits contribute to its development. Gradually, gut–brain–microbiome interactions have become regarded as the probable mechanism. Notably, the intestinal microbiome is generally considered to play an important role in IBS, including its occurrence and diagnosis. Meanwhile, probiotics and prebiotics such as purified fungal metabolites are emerging as promising therapies [3,4].

The intestinal microbiota comprises various microorganisms, including bacteria, viruses, fungi, archaea, and other parasitic microorganisms. Such a microbial community structure is believed to represent individuals and contribute to host metabolism, immunity, and even organ development [5]. Out of all the microbial organisms, the relationship between bacteria and IBS is the most well studied: bacterial alterations in IBS patients have been observed, such as an increased ratio of the phylum Firmicutes to Bacteroidetes [6,7], and at the genus level, the abundance of *Bacteroides* [8,9] increases while *Bifidobacterium* decreases [7]. Moreover, gut bacteria also affect the symptoms of IBS. Enterotoxigenic *Bacteroides fragilis* degrades intestinal glycoproteins and further affects intestinal ecology and motility to induce diarrhea and abdominal pain [10,11]. In contrast, *Bifidobacterium infantis* can improve the symptoms of IBS by suppressing proinflammatory cytokines and maintaining anti-inflammatory cytokines [12,13].

Recently, research on the IBS intestinal microbiome has been dedicated to bacteria, and only a few studies have discussed the nonbacterial microbiome in IBS [14,15,16,17]. This review aims to summarize and identify the links between nonbacterial microbiota and IBS (Figure. graphical abstract). In general, the fungal community is altered in IBS patients, and certain fungi are related to symptom severity and can serve as biomarkers. More importantly, fungi can promote the development of IBS by causing immune activation, intestinal barrier disruption, and visceral hypersensitivity via metabolites or fungi-induced responses. Their interaction with bacteria may also take part in the pathogenesis. Additionally, novel and valuable therapeutic methods are proposed, including fungicides, blocking the pathogenic pathway, probiotic fungi, fungi-related prebiotics, and fecal microbiota transplantation (FMT). Similarly, for viruses, archaea, and other parasitic microorganisms, their alterations in IBS patients and their associations with the symptoms, diagnosis and treatments of IBS are also discussed.

## 2. Mycobiome

Although fungi account for only approximately 0.1% of the intestinal microbiome and exhibit less diversity than their bacterial counterparts [18,19], they have been suggested to play an essential role in IBS. In particular, intestinal fungi dysbiosis is found in IBS-D patients who have no alterations in their intestinal bacteria, suggesting that the intestinal mycobiome is more critical in the development of IBS than bacteria [3].

Methods to study the mycobiome include ELISA (enzyme-linked immunosorbent assay) to detect certain species, culturomics, ITS (internal transcribed spacer, including ITS1 and ITS2) region-based high-throughput sequencing and shotgun metagenomics sequencing. However, each method has its own shortcomings. ELISA and culturomics only partly detect fungi. The high sequence and length variability of the ITS regions make the data analysis complex. Even shotgun metagenomics sequencing is still inaccurate for less abundant species. A modified DNA extraction method favoring the fungal community may allow us to obtain more accurate and reliable information.

### 2.1. Mycobiome Alteration in IBS

Studies have shown that the mycobiome of IBS patients is altered compared to that of healthy controls (Table 1) [3,16,20,21,22,23,24,25]. Less α-diversity [3,16,21,22] and significantly changed β-diversity [20,21] of fungi have been observed in IBS patients.

At the phylum level, the abundance of Zygomycota in IBS patients is changed, although different results have been reported [3,22]. At the genus level, the relative abundance of *Aternaria*, *Candida*, *Cladosporium*, *Debaryomyces*, *Emericella*, *Galactomyces*, *Malassezia*, *Mycosphaerella Phialemonium*, *Saccharomyces,* and *Vishniacozyma* increased in IBS patients [16,20,21], while that of *Agaricus*, *Aspergillus*, *Clavispora*, *Eurotium*, *Kazachstania*, *Pandora*, *Penicillium*, *Phoma*, *Pichia*, *Rhodotorula*, *Sporidiobolus,* and *Wallemia* decreased [3,16,20,21,22]. A change in *Mycosphaerella* has also been reported with contradictory results [3,22]. At the species level, *Candida albicans* and *C. glabrata* increased [16,21] while *Candida parapsilosis*, *Starmerella bacillaris*, *Aspergillus section Nidulantes*, *Wallemia muriae*, *Torulaspora delbrueckii*, *Rhodotorula mucilaginosa*, *Suillus luteus* [16], and *Torulospora delbrueckii* [21] decreased in IBS patients. Note that *Saccharomyces cerevisiae* has also been reported with different results [16,21].

In addition, certain fungal strains may serve as biomarkers for IBS. For instance, a model based on the four types of fungi, *Mycosphaerella*, *Aspergillus*, *Sporidiobolus*, and *Pandora*, is recognized to have a strong ability to predict IBS [3]. Based on the current evidence of mycobiome alterations in IBS patients [16], *Kazachstania turicensis*, *Monographella nivalis*, *Alternaria alternata*, and *Davidiella tassiana* may also be used to distinguish IBS patients from healthy subjects. Additionally, measuring antigens or antibodies towards valuable fungi would also facilitate diagnosis. From this perspective, the mycobiome has brought us one step closer to a noninvasive diagnosis of IBS.

### 2.2. Associations between Fungi and IBS

Fungi not only have alterations in abundance in IBS patients but are also associated with the different symptoms of them. Clinical studies have found that Zygosaccharomyces is positively associated with abdominal pain, while Malassezia is inversely associated with it [3]. For other intestinal symptoms, *Candida* positively correlates with the severity of bloating [3], while *S. cerevisiae* [26,27] and *S. boulardii* [28] have shown the potential to regulate bowel movements. The frequency of defecation has been found to correlate with reduced *Debaryomyces* and enriched *Sporidiobolus* and *Gibberella* [3]. Additionally, stool consistency seems to be inversely correlated with *Trichosporon* and *Kodamaea* [3]. Notably, whether food intolerance in IBS patients relates to the mycobiome remains unclear. One study indicates that the overgrowth of *C. albicans* may cause food intolerance, while the other suggests that there are no conclusive links [29,30].

Apart from intestinal symptoms, the mycobiome is also related to mood disorders in patients. IBS patients with psychiatric symptoms, either anxiety or depression, have distinct diversity or structure of fungi from IBS patients without psychiatric symptoms [3]. Increased *Candida* and *Wallemia* correlate with anxiety, while depression is associated with increased Zygosaccharomyces [3]. *S. boulardii* has been identified to reduce anxiety-like behavior in IBS mouse models [28].

Although the causal relationship between fungi and symptoms of IBS is unclear, many associations between them indicate that fungi more or less relate to the development of IBS. One direct and radical way to verify these findings is to transplant potential beneficial fungi, such as *S. boulardii*, *S. cerevisiae*, and *Sporidiobolus pararoseus*, into patients or models, and some of them have shown good effects [31,32]. For pathogenic fungi, measurements of fungal metabolites in breath, blood, or urine can help clarify their relationship with IBS, and utilizing germ-free mice gavaged with fungi is a direct method.

### 2.3. Potential Mechanisms by Which Fungi Promote the Development of IBS

#### 2.3.1. Metabolites

Some fungal metabolites may participate in the pathogenesis. *Candida glabrata*, which contains β-mannosides and increases in IBS patients, aggravates colitis in mice, while this impact of the β-mannoside-reduced strain is much less, suggesting that β-mannosides contribute to fungal invasion [33]. Chitin of the fungal cell wall can stimulate the host to release TNF-α and IL-6 through TLR3, 8, and 9 signaling on immunocytes [34]. Moreover, β-glucan-cell wall polysaccharides released from most fungi and candidalysin-encoded by hypha-associated gene *ece1* in *C. albicans* greatly influence intestinal homeostasis, which will be discussed later.

On the other hand, fungi influence host metabolites involved in the intestinal environment. *S. cerevisiae* worsens colitis in mice by promoting the purine metabolism of intestinal epithelia and increasing the production of uric acid [35]. Live *C. albicans* regulates tryptophan metabolism, indirectly upregulating the 5-hydroxytryptophan pathway, in mononuclear cells in vitro to promote *C. albicans* infection in mice [36].

Additionally, prostaglandin E2 (PGE2) has been reported to increase in the colon biopsies of IBS patients and contribute to intestinal inflammation by amplifying IL-17 production [37] and visceral hypersensitivity in mice [38]. Since both fungi and mast cells can produce it, further studies, such as silencing PGE2-related genes in mast cells and then detecting the PGE2 content in IBS models, may help identify the main source of it.

#### 2.3.2. Immune Activation

Low-grade immune activity in the intestine has been found in a considerable number of IBS patients, where mast cells and T cells primarily play vital roles. Mast cells in the mucosa of small [39] and large intestines [40,41] are increased in IBS patients compared to healthy controls. CD4+ and CD8+ T cells are also regarded as great contributors to IBS immune responses, although the alteration in the number of T cells in IBS is divergent [40,42,43]. Despite the similar global cytokine profiles of IBS patients and healthy subjects, some cytokine imbalances are still found in the patients. IL 1β mRNA in the rectal biopsy [44] and IL-6, IL-8 [45] and TNF-α in serum [46] increase while IL-10 mRNA in the sigmoid colon biopsies decreases [45] in IBS patients.

**Figure 1:**
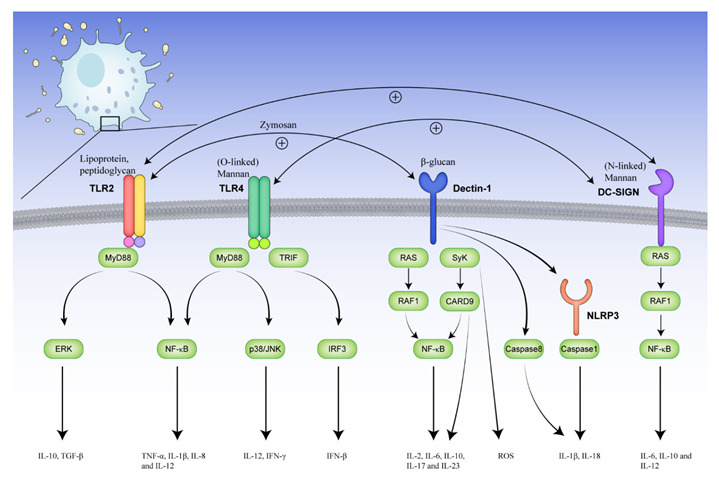
Fungi-activated receptors and the downstream signaling pathways of immune cells. TLR2 recognizes triacylated and diacylated lipoprotein and peptidoglycan of fungi to bind to MyD88, which further activates the ERK and NF-κB pathways to trigger cytokine and chemokine expression. TLR4 recognizes O-linked mannan and activates the NF-κB and p38/JNK pathways via MyD88. In addition, TLR4 induces TRIF-IRF3-mediated release of interferon-β. Dectin-1 recognizes β-glucan and triggers the phosphorylation of RAF1 via RAS proteins, ultimately stimulating the NF-κB pathway. Additionally, Dectin-1 activates the Syk-CARD9 pathway, which then leads to the production of ROS and the activation of NF-κB. Moreover, Dectin-1 also synergizes with TLR2 to recognize zymosan and enhance MAPK activation. It activates the NLRP3 inflammasome or caspase8 to induce the production of IL-1β and IL-18. NLRP3 is an intracellular receptor and forms the inflammasome with caspase1, which promotes the maturation of IL-1β and IL-18. DC-SIGN recognizes the N-linked mannan structures of fungi, and the signal activates RAF1 to secrete IL-6, IL-10 and IL-12. Moreover, by activating the same NF-κB subunit p65, DC-SIGN interacts with the TLR2 and TLR4 pathways to promote antifungal reactions. TLR: Toll-like receptor; MyD88: myeloid differentiation primary response gene 88; ERK: extracellular signal-regulated kinase; NF-κB: nuclear factor kappa B; JNK: c-JUN N-terminal kinase; TRIF: TIR-domain-containing adaptor inducing interferon-β; IRF3: interferon regulatory factor 3; RAF: rapidly accelerated fibrosarcoma; RAS: rat sarcoma; Syk: spleen tyrosine kinase; CARD9: caspase recruitment domain-containing protein 9; ROS: reactive oxygen species; MAPK: mitogen-activated protein kinase; NLRP3: NOD leucine-rich repeat and pyrin domain-containing protein 3; DC-SIGN: DC-specific ICAM3-grabbing non-integrin.

Meanwhile, fungi play a role in IBS immune activation, including mast cell and T-cell responses, via receptors, cells, and metabolites. When fungi invade the intestine, the components of the fungal cell walls, i.e., mannan, glycosylated proteins, β-glucan, and chitin [47], are the main ligands that bind to pattern recognition receptors such as TLRs [48,49], Dectin-1 [50], DC-specific ICAM3-grabbing non-integrin (DC-SIGN) [51], and NLRP3 [52] on immune cells, and activate downstream pathways to trigger cytokine and chemokine expression (Figure 1).

**Figure 2:**
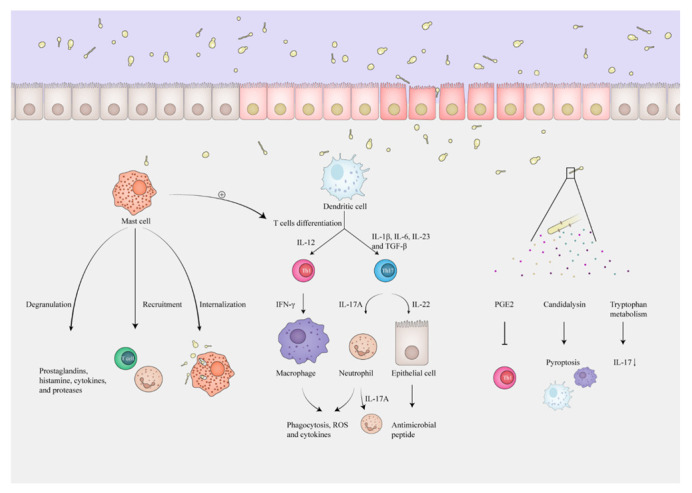
Fungi induce immune activation via various cells. Mast cells are activated by fungi to degranulate and release prostaglandins, histamine, cytokines, and proteases. In addition, they recruit T cells and neutrophils or directly internalize fungi. Activated dendritic cells and mast cells release various cytokines to promote T-cell differentiation to defend against fungi. IL-12 induces Th1 cells to secrete IFN-γ to stimulate macrophages and promote phagocytosis and the production of ROS and cytokines. IL-1β, IL-6, IL-23, and TGF-β act on Th17 cells to recruit neutrophils by IL-17A, which then can also secrete IL-17A by themselves to recruit more cells and stimulate epithelial cells by IL-22 to release antimicrobial peptides. Meanwhile, fungi produce PGE2 to inhibit Th1 responses, cause pyroptosis of macrophages and DCs by candidalysin, and regulate tryptophan metabolism to reduce IL-17. Th cell: T helper cell; DC: dendritic cell.

For immunocytes, mouse models show that the degranulation of mast cells is triggered by β-glucans of *C. albicans*, likely through the Dectin-1 pathway, to release prostaglandins, histamine, cytokines, and specific proteases (β-hexosaminidase and tryptase) [16]. Meanwhile, an in vitro study shows that mast cells have versatile and timed responses when encountering *C. albicans* [53]. Initially, mast cells degranulate to release β-hexosaminidase and reduce *C. albicans* viability. Then, they release IL-8 and recruit neutrophils. In the final response, IL-16 is released, and mast cell extracellular traps ensnare *C. albicans*, along with observed T-cell migration (Figure 2).

Another critical cell in IBS, the T cell, can also be activated by fungi, especially Th1 and Th17 cell [54]. Activated dendritic cells (DCs) and mast cells can promote T-cell differentiation via distinct cytokines. Specifically, in vivo and in vitro studies show that IL-1β, IL-6, IL-23, and TGF-β from innate immunity activate Th17 cells [55] to recruit neutrophils by IL-17A and stimulate epithelial cells by IL-22 to defend against fungi. Moreover, the loss of Th17 polarization leads to a higher risk of fungal infections, and IL-17A receptor-deficient mice are severely impaired in their ability to clear *C. albicans* [56]. In particular, a study of a *C. albicans* infection model shows that Th17 responses are reduced by blocking Dectin-2 on dendritic cells, and Th1 responses are also decreased if coupled with a lack of Dectin-1 [57]. These results reveal the importance of Dectin-1 and Dectin-2 in T-cell activation upon fungal invasion. In addition, Th1 responses can be triggered by IL-12 and then activate phagocytes by IFN-γ, which has also been reported in IBS patients [58].

Some metabolites also regulate fungi-related immunity. Fungi such as *Candida* can utilize arachidonic acid to produce PGE2, which inhibits anti-*Candida* Th1 responses [59] and enhances fungal germination. A cytolytic peptide toxin, candidalysin, can trigger the NLRP3 inflammasome [60] and result in the pyroptosis of macrophages and DCs [61,62]. Compared to heat-killed *C. albicans*, live *C. albicans* can regulate tryptophan metabolism in mononuclear cells, downregulating the L-kynurenine pathway and indirectly upregulating the 5-hydroxytryptophan pathway, to inhibit the production of IL-17 in vitro [63] and reduce resistance to *C. albicans* infection in mouse models [36]. In summary, fungi regulate intestinal immunity via various receptors, immune cells, and metabolites. The intestinal immune activities in IBS patients, such as mast cell and T-cell responses, may be partly triggered by fungi, indicating the significance of fungi in developing IBS.

#### 2.3.3. Increased Intestinal Permeability

Normally, the intestinal barrier is semipermeable and blocks the invasion of foreign pathogens to maintain homeostasis inside and outside the intestine, but intestinal permeability is increased in IBS [64,65]. For instance, IBS patients have higher paracellular permeability and lower expression of ZO-1 in the colon [66]. Fecal supernatants from IBS-D patients cause increased paracellular permeability in mice [67], indicating that increased intestinal permeability is another potential mechanism of IBS.

Fungi probably disrupt the intestinal barrier via their antigens and metabolites to contribute to the occurrence of the disease. For instance, among the fungal species, *C. albicans* has been well studied, and there is a cloned expansion of *C. albicans* isolates in IBS patients who are also more invasive and capable of producing more hyphae compared to the control subjects [21]. *C. albicans* can attack the intestinal barrier in several ways, possibly occurring in an orderly or simultaneous manner. First, *C. albicans* binds to the mucin of the intestinal wall by hydrophobic interactions and decomposes them by secreted aspartyl proteinase [68]. Next, various adhesins on the hyphae mediate direct adhesion to intestinal cells. *als3* encodes a glycoprotein that binds to cadherin on the cell membrane in vitro and promotes the endocytosis of *C. albicans* [69]. Adhesin Hwp1 forms a covalent link with intestinal cells to facilitate the invasion of the cells [70]. Then, as in Caco-2 monolayers, *C. albicans* lyses E-cadherin in the intestine by secreted aspartyl proteinase [71] and reduces the levels of occludin and ZO-1 [72]. Finally, even though *C. albicans* is endocytosed, it can escape from cells by depleting glucose inside the cells and piercing the cell membrane by its hyphae. Moreover, candidalysin damages the epithelial barrier by destroying the cell membrane or promoting pyroptosis [61,62]. Other fungi, such as *C. glabrata*, have similar virulence factors and pathogenic mechanisms [73]. Additionally, *S. cerevisiae* worsens colitis in mice by increasing intestinal permeability [35], which may result from promoting the purine metabolism of intestinal epithelia to increase the production of uric acid. Uric acid stimulates the NLRP3 inflammasome to influence intestinal stability [74].

Various factors released by antifungal immunity also increase intestinal permeability. An in vitro study indicates that increased TNF-α and IFN-γ cause tight junction (TJ) disruption by inducing myosin II regulatory light chain phosphorylation [75] and redistributing occludin, claudin 1, and claudin 4 [76]. Thus, the fungi-induced release of TNF-α and IFN-γ, which are mainly produced by macrophages and T cells, may exacerbate barrier damage in IBS patients. Active mast cells in IBS patients also relate to the disrupted barrier. Downregulated ZO-1 mRNA in IBS patients correlates inversely with mast cell-derived tryptase mRNA [77]. Meanwhile, tryptase reduces the expression of other TJ proteins by activating protease-activated receptor 2 on colonocytes [78]. In addition, enhanced cysteine proteases trigger the enzymatic degradation of occludin in patients to contribute to increased intestinal permeability [79].

Therefore, fungi cause increased intestinal permeability and simultaneously trigger body regulatory activities to modify intestinal motor, sensory, and secretory functions. Notably, *S. boulardii* improves gastrointestinal transit in IBS models [28] due to its effect on enhancing barrier function and regulating immune responses [80]. Other IBS symptoms involved in intestinal movement and secretion may also relate to the fungi. Even if the causal links between fungi and IBS have not been confirmed, the evidence on these pathophysiological alterations caused by fungi suggests that the role of the fungi in the disease cannot be ignored.

#### 2.3.4. Visceral Hypersensitivity

**Figure 3:**
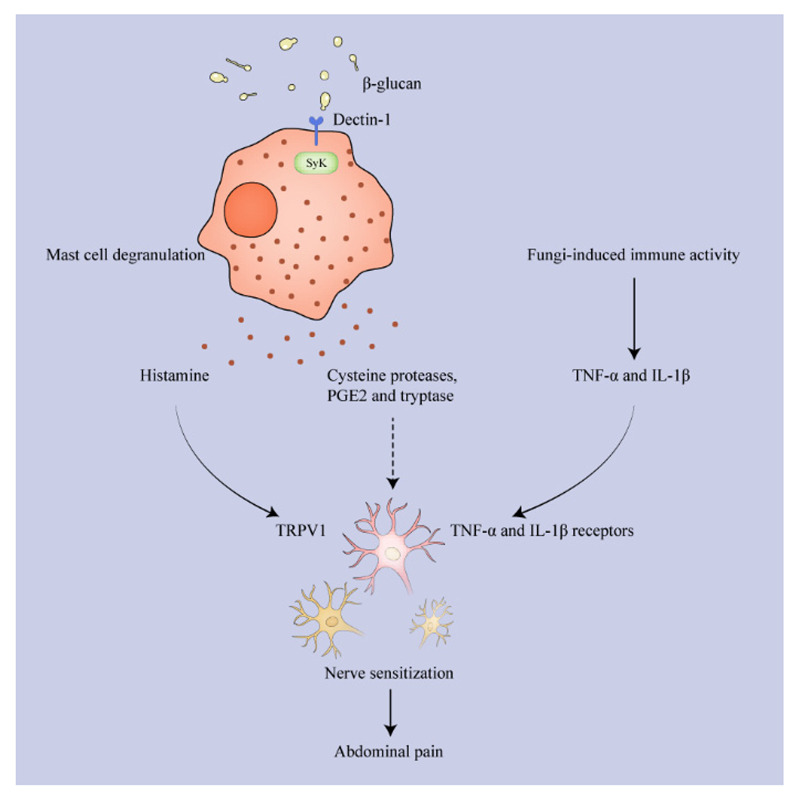
Fungi arouse abdominal pain via mast cell degranulation and other induced immune activity. β-glucan from the fungus binds to Dectin-1 to activate mast cells, which then degranulate and release histamine to stimulate TRPV1 on the nerves. Other released cysteine proteases, PGE2, and tryptase also cause nerve sensitization, but the direct mechanism is unclear. TNF-α and IL-1β from fungi-induced immune activity bind to the corresponding receptors on the nerves and finally provoke abdominal pain. TRPV1: transient reporter potential channel V1.

Abdominal pain is a common symptom of IBS patients and is closely associated with visceral hypersensitivity or nerve sensitization, and evidence has indicated an association between fungi and visceral hypersensitivity. Fungicides fluconazole and nystatin prevent the post-stress visceral hypersensitivity of IBS rats. In addition, normosensitive rats present visceral hypersensitivity when transplanted with the cecal mycobiome from visceral hypersensitive rats [16]. Moreover, β-glucan induces mast cell degranulation via the Dectin-1-Syk pathway, and a Syk inhibitor reverses visceral hypersensitivity in mice [16,81]. Therefore, β-glucan from fungi may induce abdominal pain in IBS by activating mast cells to release various neurosensitizing substances (Figure 3). The released histamine causes abdominal pain by intensifying nociceptor transient reporter potential channel V1 (TRPV1) responses of patients’ submucosal neurons, and the antagonist of histamine receptor H1 significantly improves the symptoms and quality of life of the patients [82,83]. Furthermore, cysteine proteases [79], PGE2 [38], and tryptase [84] are also enhanced in IBS patients compared to healthy controls, and they have been found to be positively associated with visceral hypersensitivity. The hypersensitivity can be prevented by their corresponding receptor antagonists, but the detailed pathways are still unknown. Moreover, visceral afferent nerves express TNF-α and IL-1β receptors, and TNF-α has been observed to effectively motivate mechanical hypersensitivity of the colon and IL-1β has increased the basal firing level of mouse colonic sensory nerves [85]. Therefore, fungi may also raise the level of pain by locally increasing cytokines in the intestine [86,87].

#### 2.3.5. Interaction between Fungi and Bacteria

As the main components of the intestinal microbiota, fungi and bacteria constantly interact with each other and are in a dynamic balance. However, fungal–bacterial correlation declines in IBS patients compared to the healthy subjects [3,22]. In addition, *Candida* has negative correlations with the abundance of certain fecal bacteria in healthy subjects, while in IBS-D patients, all these correlations turn positive [3]. Moreover, the therapeutic effect of rifaximin on IBS may be due to the enhanced relations between fecal bacteria and fungi in IBS patients [22]. These results indicate that the interaction between fungi and bacteria participates in the pathogenesis of IBS.

Unfortunately, no detailed reports explicitly address the fungal–bacterial interaction in IBS, but studies concerning intestinal disorders can provide some enlightenment. Correlations between fungi and bacteria underlie the development of colitis, which is worsened by *C. albicans* and improved by *S. boulardii* [88]. Broad-spectrum antibiotic treatment prevents both effects, while supplementation with Enterobacteriaceae reestablishes them, indicating that bacteria are essential for fungi to function [88]. A study of the human gut microbial metabolome shows that intestinal commensal bacteria inhibit the growth of opportunistic yeasts by blocking the fungal growth-related targets of the rapamycin pathway [89]. Moreover, indoles from the tryptophan metabolism of the symbiotic bacteria balance mucosal and barrier homeostasis and prevent the colonization of *Candida* via the aryl hydrocarbon receptor-IL 22-related pathway [90,91]. Notably, as a common probiotic for IBS patients, Lactobacillus blocks *C. albicans* from adhering to epithelial cells by their exopolysaccharides [92], prevents hyphal orphogenesis by metabolized short-chain fatty acids [93,94] and degrades chitin structures by chitinase [95], indicating that its beneficial effects for IBS may partly result from the repression of *Candida*.

Meanwhile, fungi also affect bacteria in the intestine. *Debaryomyces hansenii* restores the density and diversity of intestinal bacteria to promote intestinal balance in an antibiotic-induced diarrhea mouse model [96]. *C. albicans* facilitates the colonization of pathogenic *Enterococcus faecalis* while it counters probiotic *Lactobacillus* spp. [97], although the mechanism is not well known.

On the whole, the interaction between fungi and bacteria takes part in intestinal homeostasis, but more studies should be carried out to explore its influence on IBS to guide microbiota adjustment therapies.

### 2.4. Fungi-Related Treatment

Currently, the treatment for IBS includes dietary therapies, μ-opioid agonists, antispasmodics, laxatives, probiotic bacteria, etc. [4], but their efficacy remains unsatisfactory. With the high prevalence of IBS, more novel therapeutic strategies are in demand. Based on the alteration and the potential pathogenic mechanism of fungi in IBS, novel and valuable treatments, such as fungicides, pathogenic pathway blockage, probiotic fungi, fungi-related prebiotics and FMT, have emerged and are expected to solve the current issue.

#### 2.4.1. Using Fungicides or Blocking the Fungal Pathogenic Pathways

Fungicides effectively clear fungi and benefit patients. An analgesic effect of the fungicides fluconazole and nystatin has been observed when treating maternal separation rats [16]. Miltefosine relieves visceral hypersensitivity in rat models [98] and modulates the fecal mycobiome by inducing metacaspase-dependent apoptosis [99,100]. However, due to broad-spectrum antimicrobial activity and unpredictable side effects, fungicides are usually not accepted as conventional treatments and are only recommended when certain clear indications are present [101].

To reduce the potential side effects and interference with the intestinal environment, targeting fungal pathogenic pathways, especially fungal adherence and invasion, is a promising option for treatment. IgA, which targets and suppresses adhesins Als1 and Als3 of *C. albicans*, has been detected in human feces [102]. Notably, anti-*Candida* vaccines designed for these adhesions have been produced. The vaccine increases Als3-specific IgA and IgG and prevents *C. albicans*-associated damage in colitis [102], which indicates that the vaccine could be used to cautiously treat IBS patients under close follow-ups. Moreover, there are other potential targets of the treatment, such as the transcription factors Ahr1 and Tup1, which are necessary for the expression of als3 [103]. Inhibiting fungal invasion or overgrowth is another strategy. The *tpk2* and *efg1p* genes govern invasion and hyphal formation [104,105], while farnesol, the quorum-sensing molecule secreted by *C. albicans*, inhibits hyphae and biofilm formation [106,107]. Interestingly, shikonin, a plant extract for wound treatment, can inhibit the hyphal formation of *C. albicans* and reduce the fungal burden with downregulated levels of Ece1, Hwp1, Als1, and Als3 and upregulated production of farnesol [108]. Thus, these loci can be novel targets of treatment that maximally defend against pathogenic fungi and avoid affecting other microbiota.

#### 2.4.2. Probiotic Fungi, Fungi-Related Prebiotics, and FMT

Taking probiotic fungi to induce a relatively slight but favorable microbiota shift is also suitable for IBS patients. *S. boulardii* has been widely used in diarrheal diseases to enhance barrier function and mucosal immune responses [80]. For IBS patients, it also ameliorates their quality of life and abdominal pain by decreasing TRPV1 expression [28] and improving the cytokine profile in the blood and upper rectum [31]. Meanwhile, its benefits may also result from the antagonistic effect on *C. albicans* colonization [109] and the protection of the intestinal barrier, promoting the expression of E-cadherin and its redistribution to the cell surface [110]. In addition, *S. boulardii* improves gastrointestinal transit [28] and reverses anxiety behavior in the IBS model [28], but whether these effects similarly act in patients deserves more study.

*S. cerevisiae* is another commonly reported fungal probiotic for IBS that significantly alleviates abdominal pain, stool consistency and bloating in patients [27,32,111,112]. Interestingly, *S. cerevisiae* has great degradation properties on fermentable oligosaccharides, disaccharides, monosaccharides, and polyols, further revealing its ability to improve bloating [113,114]. Meanwhile, a study reports that a distinct strain of *S. cerevisiae* with genetic and phenotypic variability has pathogenic effects on the intestine [35]. Thus, the exploration of fungi needs to be more detailed and go deeper into the genetic and phenotypic levels.

Apart from probiotics, prebiotics from fungal metabolites or materials are also therapeutic methods. As discussed above, fungal glucan can induce immune responses, whereas much evidence shows that fungal glucan extracts relieve intestinal dysfunction by decreasing proinflammatory factors and colonic mucosal damage in colitis [115,116]. For IBS patients, a mixed treatment containing β-glucan is capable of relieving bloating and abdominal pain [117,118]. In IBS models, β-glucan treatment suppresses restraint stress-induced fecal pellet output and visceral pain [16,119]. Overall, glucan extracts show great potential in treating IBS patients. In addition, a combination of essential oils from menthol and carvone could be the prebiotic for IBS, which reverses visceral hypersensitivity and simultaneously alters fungal composition in maternally separated rats [24]. Nevertheless, the active ingredient and specific pathway should be determined with further research.

FMT is a method of copying intestinal microbiota composition from healthy donors to recipients and has been used to treat IBS patients, especially moderate to severe patients. Some studies show that the improvement rates of symptoms and quality of life in the FMT group are higher than those in controls [120,121] while a study finds that those in the control group are better [122], indicating that stronger evidence for the treatment is still needed. In addition, as transplantation is a mixture of bacteria, fungi, and other microbiota, it is difficult to determine the role of fungi in it. An FMT trial of *Clostridium difficile* infection suggests that there are more *Saccharomyces* and *Aspergillus* in the intestines of successful FMT recipients but more *Candida* in that of nonresponders [123]. Similar research on the intestinal mycobiome of IBS recipients after FMT trials will further show the meaning of fungi in the treatment and the disease.

In summary, fungicides, blocking the fungal adherence and invasion of the intestine, probiotics, prebiotics, and FMT can be novel therapies for IBS. The combination of these therapies may have more significant effects, but the administration should be personal and must be based on detailed intestinal microbiota information. However, these treatments lack the validation of clinical data, and more randomized controlled trials are needed to determine their efficacy and possible side effects.

## 3. Virome

Developing metagenomics studies have revealed that viruses commonly appear in the microbiota of healthy humans. There are approximately 10^9^ viral particles per gram of feces [124]. Classified by host types, the virome is composed of bacteriophages/phages (97.7%), eukaryotic viruses (2.1%), and archaeal viruses (0.1%) [125,126]. Unfortunately, thus far, many viruses remain unclassified due to the lack of universal viral markers and knowledge about their specific hosts or infection activities. Moreover, as the content of the existing virus database is very limited, the majority of reads in metagenomics studies cannot be well annotated [127,128]. Hence, existing studies may have overlooked the importance of viruses in the pathophysiological process.

### 3.1. Virome Alteration in IBS

Emerging evidence suggests that viruses contribute to some intestinal diseases; for instance, the intestinal virome has disease-specific alterations in IBD patients [129,130]. However, only a few studies have reported its alterations in IBS. The α-diversity of the virome in IBS patients is lower than that in controls, and β-diversity is significantly different between IBS patients and controls [17,131].

There are alterations in the abundance of certain viruses in IBS patients (Table 2). At the genus level, three unclassified viruses from the families Mimiviridae, Podoviridae, and Siphoviridae increased, while four unclassified viruses from the same three families decreased in IBS patients [131]. More precise detection of these viral genera will reveal their alteration and the possible association with IBS. At the species level, *Pandoravirus salinus* significantly increased in IBS patients, while *Choristoneura biennis entomopoxvirus*, *Aureococcus anophagefferens virus*, *Phaeocystis globose virus*, *Pandoravirus inopinatum*, and some other unclassified species decreased in the patients [17]. Additionally, a study reported the difference in the virome between IBS subtypes. *Lactobacillus virus* increased in IBS-C patients compared with IBS-D patients, and distinct but unclassified species of the families Microviridae, Myoviridae, Siphoviridae, and Podoviridae were significantly changed in IBS-C or IBS-D patients that need further research [132].

In summary, due to the limited virus database and the lack of precise detection methods, virome alteration in IBS is less known and still in its infancy. It is necessary to increase the research projects on it and develop novel methods with higher sensitivity and specificity to reveal the alteration of viruses in IBS and its influences.

### 3.2. Association between Viruses and IBS

Although research on the virome in IBS is limited, some reports have revealed that certain viruses can influence the development of IBS. Norovirus is now considered a major cause of gastroenteritis and is closely related to postinfectious IBS (PI-IBS). Studies have shown a higher prevalence of PI-IBS after this viral gastroenteritis [133,134] and its symptoms are mild, IBS-like, and persistent, with low detectable titers of virus for 8 weeks or even a year [135]. The possible mechanism may be related to enterocyte apoptosis, acceleration of cell turnover, and widened intercellular spaces. Moreover, the villous surface area is reduced by 50% with mononuclear cells infiltrating the lamina propria [136,137]. Thus, the occurrence of IBS may be due to residual viruses or pathophysiological changes, but the causal relationship has not been well explained.

Severe acute respiratory syndrome coronavirus-2 (SARS-CoV-2) is also related to IBS [138,139]. Patients with diarrhea tend to have a higher viral RNA load [140] and the virus has been detected inside enterocytes and fecal samples [141,142]. Perhaps the viral spike protein binds to angiotensin-converting enzyme 2 and allows the virus to enter the host cells [143,144]. Compared to the controls, the levels of fecal IL-8 and IL-23 are higher, while IL-10 is lower in COVID-19 patients, suggesting that the gastrointestinal tract is immunologically active during infection [140]. More attention should be given to the association between SARS-CoV-2 and IBS.

A high prevalence of IBS has also been shown in other virus-infected people. Chronic hepatitis C virus (HCV) patients usually complain of abdominal pain, discomfort, and functional nausea [145,146]. A study including 454 individuals showed that the proportion of IBS patients in the HCV group (66%) was significantly higher than that in the HBV group (22%) and healthy subjects (18%) [147]. In addition, increased Herpesviridae is detected in IBS patients, yet no association between Herpesviridae and IBS is definite. Herpesviridae is also increased in IBD patients [148] and can activate human endogenous retroviruses in the colon [149], indicating its potential to induce chronic inflammation.

Studies have shown that viruses can regulate the host immune state by TLR (TLR3, 7, 8 and 9) [150,151,152] and RIG-1-like receptor (RIG-1 [153], MDA5 [154], and LGP2 [155]) pathways. Notably, a recent study explored the relationship between the virome and colonic gene expression in IBS patients [132]. Most virus-related host genes are associated with infection and immune responses, such as CD4, TLR2, interleukins (IL2RG, IL3RA, IL6R), and human leukocyte antigens (HLA-DMB, HLA-DPA1, HLA-DRA). In addition, the genes related to the zinc-finger motif, cell-cell contact or epithelial barrier function are associated with a group of phages from the families Podoviridae, Microviridae, and Siphoviridae. In summary, few studies have shown links between viruses and IBS, while evidence indicates that they are undiscovered but not nonexistent. These findings regarding viruses provide more ideas for further research to reveal their significance in IBS.

### 3.3. Viral Treatment for IBS and Future Directions

A cocktail of phages is reported to relieve colitis symptoms in a mouse model [156], but little is known about the viral treatment for IBS. However, reducing *Lactobacillus virus* is a potential target for IBS treatment. As *Lactobacillus virus* increases in IBS-C [132] and its host, *Lactobacillus brevis*, has been used to improve the quality of life of IBS patients [157,158], reducing the virus may be another way to show the beneficial effect.

Additionally, viruses can regulate the immune state to benefit IBS patients. After acute herpesvirus infection, the virus enters a chronic latency situation, which can persist throughout the host’s life. It increases the level of IFN-γ and systemically activates macrophages to maintain a better immune state [159], which is a reestablished symbiotic homeostasis between the host and the virus. Meanwhile, IFN promotes epithelial regeneration after colitis [160] and radiation-induced [161] intestinal injury in a mouse model, suggesting that mildly increasing IFN can benefit IBS patients. However, these treatments will also bring possible side effects of excessive immune activation or cytokine storms. Therefore, longitudinal metagenomic research and clinical trials are required to assess their potential benefits and losses.

To date, much work about the virome in IBS still needs to be done. First, it is vital to develop more accurate and convenient sequencing technology and improve the enterovirus annotation database to better identify and analyze the viruses. Second, the association between viruses and the host in IBS patients is vague, but it is worth further exploring whether and how the viruses arouse IBS symptoms. Finally, further understanding and utilizing the interaction between viruses and bacteria or fungi is of value in restoring intestinal homeostasis of IBS patients, such as lysing pathogens and transferring antipathogen properties to commensal bacteria by certain viruses.

## 4. Archaeome

Archaea are originally found in various extreme natural ecosystems and represent the limits of life on this planet. However, they can also live in a gentle environment, including oceans, marshlands, and paddy fields, and be stable commensals of skin and human digestive tracts [162,163]. Although archaea only account for a small fraction of the intestinal flora (0.05–0.8%), it has been gradually found that they are involved in immune modulation, methanogenesis, trimethylamine metabolism, and transformation of heavy metals [18,162,164]. Methanogens are the main archaea (approximately 90%) in the human gastrointestinal tract [165], which physiologically convert hydrogen, acetic acid, or methyl compounds into methane. Genus *Methanobrevibacter* and *Methanosphaera* of order Methanobacteriales are the predominant methanogens in the human intestine [162]. Methanogens, mainly in the colon, have stable biofilm and their ability to acquire the nitrogen nutrients is much stronger than that of other microbes. At present, detection methods of archaea mainly include cultivation, qPCR, and 16S rRNA sequencing, but there are no specific protocols or primers targeting archaea [166,167]. Metagenomic sequencing would be a better choice, but it is also not accurate or specific enough. Limited detection methods make it challenging to research archaea.

### 4.1. Archaeome Alteration in IBS

More evidence shows that the abundance of *Methanobrevibacter smithii* in IBS patients is higher than that in healthy subjects (Table 3) [9,168,169,170,171], while other studies report decreased abundance of *Methanobrevibacter*, Methanobacteriaceae, and Methanobacteria in the patients [7,15,172,173,174] or suggest no difference in abundance of Methanobacteriales between IBS and the controls [9]. Notably, most studies support that methanogens play a greater role in IBS-C than IBS-D as more IBS-C patients have Methanobacteriales [9] and *Methanobrevibacter* [7] than IBS-D patients [7]. Copies of *M. smithii* in IBS-C are significantly greater than those in IBS-D [170]. In general, methanogens seem to play a role in IBS, especially IBS-C, but this needs to be verified in larger populations.

### 4.2. The Effect of Methanogens on IBS

To date, studies of the association between archaea and IBS have mainly focused on methanogens, but whether they are beneficial or detrimental remains unclear [175,176,177]. In fact, methanogens often present a symbiotic relationship with bacteria. Hydrogen gas usually originates from the fermentation of bacteria, but its accumulation suppresses bacterial energy production. Therefore, hydrogen removal by methanogens enhances the degradation of organic material, metabolic activity of bacteria, and energy absorption in the intestine [178,179]. Alternatively, some methanogens can also convert acetate or methanol/methylamines into methane to participate in the metabolism of the intestinal microenvironment. Thus, archaea interact with the host and other microbes to maintain intestinal homeostasis, but when the balance is broken, methanogens may aggravate intestinal disorders to potentially cause IBS.

The results of quantitative PCR indicate that methanogens are positively related to constipation and bloating [170,180,181], which is in line with other studies that use the lactulose breath test to detect methanogens [9,181,182,183]. Further investigation demonstrates that methane slows intestinal transit by enhancing the contractile activity of the intestinal circular muscle and may lead to the constipation of IBS-C [184,185], while other research suggests that lower Methanobacteriales and exhaled methane are associated with severe IBS patients [9]. Hence, archaeal function needs to be further explored in large-scale experiments.

### 4.3. The Utilization of Methanogens in IBS

Methane is not utilized by humans and is discharged through the anus (80%) and breath (20%). Consequently, the measurement of methane in respiration can reflect the quantity and physiological activity of live methanogens [169,170,186]. Furthermore, the test has been recommended for patients with clinical constipation or slow gastrointestinal transmission [187] and to assist in the identification of IBS-C [9,183,188].

Additionally, targeting methanogens is another potential treatment for IBS, especially IBS-C. Neomycin combined with rifaximin reduces breath methane and effectively improves constipation, bloating, and straining in IBS-C [189]. Statins, such as lovastatin and mevastatin, block the biosynthesis of the methanogen membrane by inhibiting HMG-CoA reductase [190] and directly prevent methanogenesis by occupying the binding sites of methanogenic enzymes [191,192]. In addition, the lactone form of lovastatin inhibits the growth of *Methanobrevibacter* [193]. As such, statins are considered a potential treatment to improve constipation in IBS-C.

## 5. Other Parasitic Microorganisms

Apart from fungi, viruses, and archaea, the human intestinal microbiota also includes other parasitic microorganisms, such as *Blastocystis* and *Dientamoeba*. Parasitic microorganisms are also involved in the pathophysiology of IBS, especially PI-IBS. It has been reported that PI-IBS accounts for 6–50% of all IBS cases and can even be caused by an infection occurring several years ago [194,195].

Many parasitic microorganisms have an association with IBS. People infected with *Blastocystis hominis* [196], *Giardia lamblia* [197], *Cryptosporidium hominis*, *Cryptosporidium parvum* [198,199] and *Trichinella* [200] have a higher risk of IBS, and IBS patients have a higher *B. hominis*-positive rate than controls [201,202]. In addition, *B. hominis*- and *Dientamoeba fragilis*-infected patients exhibit a variety of IBS-like symptoms, such as diarrhea, cramps, and abdominal pain [196,203]. A third of cryptosporidiosis patients infected by *C. hominis* or *C. parvum* have had persistent diarrhea and abdominal pain for 12 months [199].

Some potential mechanisms of these parasitic microorganisms have been reported. *G. lamblia* infection damages intestinal TJs, promotes mucosal adherence, invasion, and translocation of bacteria [204], and suppresses the immune defense of epithelial cells [205,206]. Moreover, *G. lamblia*-infected patients have impaired 5-HT release, increased duodenal cholecystokinin cells and higher plasma cholecystokinin levels [207,208], causing delays in gastric emptying and postprandial colonic contractions [209,210]. Additionally, continuous antigenic exposure of *Blastocystis* leads to low-grade inflammation and increased intestinal permeability [136]. *D. fragilis* causes inflammation by inducing intestinal infiltration of eosinophils, neutrophils, and lymphocytes [211].

In addition, some parasitic microorganisms can be actively utilized to build IBS models. The *Trichinella spiralis* model is the most common one, with the characteristics of intestinal hypersensitivity, hypercontractility, alteration in secretion, and sensory neuron activation [212,213,214]. The *Nippostrongylus brasiliensis* model exhibits motility dysfunction and intestinal hypersensitivity without mechanosensitivity [215]. The *C. parvum* model shows jejunal hypersensitivity, intraepithelial lymphocyte infiltration, and mast cell hyperplasia [216], but other features of IBS are unidentified. In conclusion, the infection or increase in some parasitic microorganisms probably causes the development of IBS.

## 6. Conclusions

Mounting evidence from preclinical and clinical studies has confirmed the significance of intestinal microbiota for IBS, which should be reasonably viewed as an ecological system of bacteria, fungi, viruses, archaea, and other parasitic microorganisms. Meanwhile, more attention should be paid to the previously ignored nonbacterial microbiome, which is involved in the diagnosis, pathogenesis, and treatment of IBS. Less fungal and viral diversity and some alterations in mycobiome, virome, and archaeome have been found in IBS patients, such as increased *C. albicans*, *C. glabrata*, *Pandoravirus salinus*, and *M. smithii*. Their alterations in IBS patients suggest that they can contribute to noninvasive diagnosis. For instance, certain fungi and methanogens can be biomarkers of the disease. More importantly, the nonbacterial microbiome is related to the symptoms and participates in the pathogenesis of IBS via immune activation, increased intestinal permeability, visceral hypersensitivity, and interaction with bacteria. Metabolites including β-mannosides, chitin, β-glucan, candidalysin, and PGE2 possibly play little-known but important roles in them. Additionally, novel therapies based on these findings will aid in the treatment of IBS. Fungicides, blocking fungal pathogenic pathways, probiotic fungi, fungi-related prebiotics, and FMT are potential therapies. Regulating the microbial community structure and immune state by certain viruses are also promising therapeutic methods. Meanwhile, reducing methanogens and clearing parasitic microorganisms possibly benefit patients. Thus, a more holistic attitude towards the microbiome in IBS and well-designed randomized controlled trials are needed to convert these inspiring findings into potent therapies so that patients and even healthy individuals can benefit from the achievements.

Notably, specific microbial components can be purified and then used to treat patients. For instance, the chitin of *S. cerevisiae* can train human monocytes to enhance their immunity to bacteria or fungi [34]. Human monocytes exposed to pure chitin or chitin-containing fungi such as *S. cerevisiae* in advance show more production of TNF-α and IL-6 than the controls under the stimulation of lipopolysaccharide or fungal pathogens such as *C. albicans*. In mice with systemic *C. albicans* infection, treatment with chitin reduces the fungal burden in the liver and increases survival rates. Similarly, pure β-glucan extracts from *C. albicans* display anti-inflammatory properties and decrease the colonization of *C. albicans* in colitis mice [217], suggesting that moderate exposure to the purified molecules may be a promising therapeutic method for IBS.

Although some progress has been made, research on the intestinal nonbacterial microbiome is still at an early stage, and its specific role in IBS remains to be further investigated. First, the detection methods of these intestinal microorganisms are not accurate or reliable. Even shotgun metagenomics sequencing cannot cover all microorganisms, in which fragments of less abundant microorganisms can be easily overwhelmed by host parts or main species. Thus, increasing the flux of detection or separating the host parts and main species in advance may improve its efficiency and accuracy. Second, studies on the mechanisms lack direct or in vivo evidence, and most potential treatments have not passed clinical trials and cannot be immediately available for clinical management. Therefore, multilevel research and clinical practice are needed to solve these issues. Last, most studies do not analyze the relationship between nonbacterial microbiota and distinct subtypes of IBS. Since the main symptoms and treatment of the subtypes are different, the effect of the nonbacterial microbiota may also be subtype-specific. Large-scale research containing distinct subtypes is required and the recruitment can be via community contacts, specialist clinics, and social media. In summary, studies of the association between nonbacterial microbiome and IBS are far from sufficient, and there is still a long way to go.

Going forward, it is imperative to incorporate all parts of the intestinal microbiota to explore their effects on the host. This review provides a critical view of how the nonbacterial microbiota affects IBS and a novel dimension of noninvasive diagnosis and precise treatment of this disease.

## Figures and Tables

**Table 1:** Alteration of fungi in irritable bowel syndrome patients.

Groups	Comparision	Change of Gut Microbiota		Potential Mechanisms	Conclusion	Method	Reference
		Increased	Decreased				
Hypersensitive IBS (n = 19) Normosensitive IBS (n = 20) Healthy controls (n = 18)	Hypersensitive IBS vs. healthy subjects	*Saccharomyces cerevisiae*, *Candida albicans* (*Kazachstania uricensis*, *Monographella nivalis*, *Alternaria alternata*, etc.) ^1^	*Phoma*, *Aspergillus section Nidulantes*, *Wallemia muriae*, *Torulaspora delbrueckii*, *Rhodotorula mucilaginosa*, *Suillus luteus*, (*Penicillium spinulosum*, *Kazachstania telluris*, *Davidiella tassiana*, etc.) ^1^	Host recognition of fungi via the Dectin-1/Syk signaling pathway causes post-stress visceral hypersensitivity.	Gut fungi is a direct cause of abdominal pain in rat maternal separation model. Fungicide treatment and fecal transplantation can reverse and restore visceral hypersensitivity.	ITS1	Sara Botschuijver [16]
	Normosensitive IBS vs. healthy subjects	*Saccharomyces cerevisiae* and *Candida albicans* (*Candida solani*, *SporoboIomyces roseus*, *Candida tropicalis*, etc.) ^1^	*Phoma*, *Aspergillus section Nidulantes*, *Wallemia muriae*, *Torulaspora delbrueckii*, *Rhodotorula mucilaginosa*, *Suillus luteus*, (*CystofiIobasidium infirmominiatum*, *Candida glaebosa*, *CyberIindnera C f. jadinii*, etc.) ^1^				
	Hypersensitive IBS vs. normosensitive IBS	(*CyberIindnera c.f. jadinii*, *Sporobolomyas roseus*, *Penicillium commune*, etc.) ^1^	(*Candida solani*, *Botryotinia fuckeliana*, *GIoeophyiIum trabeum*, etc.) ^1^				
IBS (n = 20) Healthy subjects (n = 18)	IBS vs. healthy subjects	Class Saccharomycete, (genus *Candida*, *Galactomyces*, *Candida glabrata*) ^2^	Cultivable fungal diversity, (genus *Pichia*, *Aspergillus*, *Rhodotorula*, *Penicillium*; *Torulospora delbrueckii*, *Starmerella bacillaris*, *Candida parapsilosis*, *Saccharomyces cerevisiae*) ^2^	*Candida* spp. isolates from IBS display distinct peculiar virulent and invasive traits.	The gut mycobiota may be involved in IBS and contribute to intestinal hypersensitivity.	ITS1, Culturomics ^2^	Piero Sciavilla [21]
IBS patients (n = 80; IBS-C: n = 30, IBS-D: n = 21 and IBS-M: n = 29) Control subjects (n = 64)	IBS vs. controls	Genus *Candida*, *Malassezia*, *Cladosporium*, *Mycosphaerella*, *Vishniacozyma*, *Aternaria*	Genus *Agaricus*, *Kazachstania*, *Clavispora*, *Rhodotorula*	Not mentioned	Mycobiome may identify clinically important disease if used in combination with the bacteriome and metabolome.	ITS1	A. Das [20]
	IBS-C vs. IBS-D vs. IBS-M	No significant difference	No significant difference				
IBS-D (n = 55) Healthy controls (n = 16)	IBS-D vs. healthy controls	Genus *Phialemonium*, *Emericella*, *Debaryomyces*, *Saccharomyces*	Phyla Zygomycota; genus *Mycosphaerella*, *Aspergillus*, *Sporidiobolus*, *Pandora*, *Eurotium*, *Wallemia*	Not mentioned	Fungi were more susceptibly altered than gut bacteria in D-IBS. Certain fungal genera were identified to differentiate D-IBS from HC.	ITS2	Gaichao Hong [3]
IBS (n = 269) Control subjects (n = 277)	IBS vs. control subjects	IgG antibodies against *Candida albicans*		Sugar seems to be a confounder in the context of the *Candida albicans* IBS association and facilitate the growth of *Candida albicans*.	There are higher levels of IgG antibodies against *Candida albicans* in the IBS and positive correlation between severity of symptoms and IgG antibodies against *Candida albicans*.	ELISA	Solveig C Ligaarden [23]
IBS-D (n = 30) Health (n = 19)	IBS-D vs. healthy subjects	Phyla Zygomycota, genus *Mycosphaerella*	α-diversity, genus *Aspergillus* and *Sporidiobolus*	Not mentioned	There is obvious distinction in fecal fungal structure between health and IBS.	ITS2	Ying Li [22]
Hypersensitive maternally separated rats (n = 8) Normally sensitive rats (n = 9)	Hypersensitive maternally separated rats vs. normally sensitive rats	Fungal α-diversity at species level, class Dothideomycetes, Sordariomycetes	Class Wallemiomycetes	Menthol activates TRPM8 to desensitize TRPV1 involving in visceral pain perception.	Essential oils modulate the in vivo mycobiome to reverse the post-stress visceral hypersensitivity.	ITS1	Sara Botschuijver [24]
Improved IBS patients (n = 8) Unimproved IBS patients (n = 8)	Improved IBS patients vs. unimproved IBS patients	-	-	-	No association of the mycobiota with improvement of sensitivity in IBS but *C. albicans* strains show both inter- and intraindividual genomic variations.	ITS1	Isabelle A. M. van Thiel [25]

Comparison: A vs. B: Increased signifies an increase in condition A relative to condition B; decreased signifies a decrease in condition A relative to condition B. ^1^ Using another approach called the elastic net classification model to address mycobiome data by the study. ^2^ Culturomics: Isolation and identification of fungal cultivable single-cell pure colonies from stool samples. Species in brackets are detected by culturomics. ITS: internal transcribed spacer; IBS-C: IBS with constipation; IBS-D: IBS with diarrhea; IBS-M: IBS with mixed bowel habit; ELISA: enzyme-linked immunosorbent assay; TRPM8: transient receptor potential ion channel melastatin subtype 8; TRPV1: transient receptor potential cation channel subfamily V1.

**Table 2:** Alteration of viruses in irritable bowel syndrome patients.

Groups	Comparison	Change of Gut Microbiota		Potential Mechanisms	Conclusion	Method	Reference
		Increased	Decreased				
IBS (n = 25) Healthy controls (n = 17)	IBS vs. healthy controls	*Pandoravirus salinus*	Order Megavirales, *Centapoxvirus Unclassified* ^1^ *Orthopoxvirus Unclassified* *Choristoneura biennis entomopoxvirus* *Capripoxvirus Unclassified* *Prymnesiovirus Unclassified* *Chlorovirus Unclassified* *Aureococcus anophagefferens virus* *Phaeocystis globosa virus* *Pandoravirus inopinatum* *Adenoviridae Unclassified* *Ligamenvirales Unclassified* *Rudiviridae Unclassified*	Not mentioned	Viral taxa can be used as a diagnostic biomarker or anti-viral drugs for the treatment of IBS.	Metagenomics analysis	Mina Hojat Ansari [17]
IBS (n = 55) Control (n = 51)	IBS vs. controls	1 VC ^2^ in family Mimiviridae, 1 VC in family Podoviridae, and 1 VC in family Siphoviridae	1 VC in family Mimiviridae, 1 VC in family Podoviridae, and 2 VC in family Siphoviridae	Not mentioned	The gut virome in IBS differs from that of controls, which can facilitate development of new therapeutics.	Metagenomic sequencing	Coughlan, S. [131]
IBS-C (n = 17) IBS-D (n = 17) Control (n = 16)	IBS-D vs. controls	1 specie of family Microviridae, 1 Myoviridae, and 1 Podoviridae	-	Not mentioned	Gut virome is stable over time and affected by diet. It influences host function via interactions with gut bacteria and/or altering host gene expression.	Metagenomics sequencing of the VLP	Kathie A Mihindukulasuriya [132]
	IBS-C vs. controls	2 Microviridae, and 1 Siphoviridae	-				
	IBS-D vs. IBS-C	1 Microviridae, 1 Myoviridae, 1 Siphoviridae, and 2 Podoviridae	3 Microviridae, 1 Myoviridae				

Comparison: A vs. B: Increased signifies an increase in condition A relative to condition B; decreased signifies a decrease in condition A relative to condition B. ^1^ Unknown species that are unclassified of the relevant genus. ^2^ VC (viral clusters): Nonredundant contigs with viral signals in the sequencing study, analogous to the genus/subfamily taxonomic level. VLP: viral-like particles.

**Table 3:** Alteration of archaea in irritable bowel syndrome patients.

Groups	Comparison	Change of Gut Microbiota		Potential Mechanisms	Conclusion	Method	Reference
		Increased	Decreased				
IBS (n = 110) Healthy controls (n = 39)	IBS vs. healthy controls	No difference in Methanobacteriales		Not mentioned	IBS symptom severity is associated with exhaled CH4 and the presence of Methanobacteriales.	Quantitative PCR ^1^	Julien Tap [9]
IBS without treatment (n = 44) Healthy participants (n = 66)	IBS vs. healthy controls		Methanobacteria	Sulfate-reducing bacteria does not compete with the Methanobacteria.	IBS-M and IBS-D patients are characterized by a reduction of Methanobacteria, with excess of abdominal gas.	16S rRNA	Marta Pozuelo [15]
IBS-C (n = 20) IBS-D (n = 20) IBS-M (n = 7) Healthy controls (n = 30)	IBS vs. healthy controls	*Methanobrevibacter smithii*		Not mentioned	Patients with IBS, particularly IBS-C, had higher *M. smithii* than HC.	Quantitative RT-PCR ^2^	Ujjala Ghoshal [170]
IBS-C with methane >3 ppm (n = 9) IBS without breath methane (n = 10)	IBS-C with methane >3 ppm vs. IBS without breath methane	*Methanobrevibacter smithii*		Not mentioned	The number and proportion of *M. smithii* in stool correlate well with amount of breath methane.	Quantitative-PCR ^3^	Gene Kim [171]
IBS (n = 62) Health (n = 46)	IBS vs. healthy controls		Genus *Methanobrevibacter*	Not mentioned	Not mentioned	Quantitative PCR and phylogenetic microarray	Mirjana Rajilić-Stojanović [7]
IBS-C (n = 14) Health (n = 12)	IBS-C vs. healthy controls		Methanogens	Not mentioned	There is functional dysbiosis in the gut microbiota of IBS-C.	Anaerobic culture and detection of specific activity	Chassard C [172]
IBS patients (n = 12) Healthy volunteers (n = 6)	IBS vs. healthy controls		family Methanobacteriaceae, genus *Methanobrevibacter*	Not mentioned	IBS patients have dysbiosis.	Shotgun metagenomic sequences	Adam Edwinson [174]

Comparison: A vs. B: Increased signifies an increase in condition A relative to condition B; decreased signifies a decrease in condition A relative to condition B. ^1^ Using primers targeting Methanobacteriales. ^2^ Using primers targeting *Methanobrevibacter smithii*. ^3^ Using a specific rpoB gene primer to detect Methanobacteriales. PCR: polymerase chain reaction; RT-PCR: reverse transcription-polymerase chain reaction.

## Data Availability

The data presented in this study are available in this article.

## Abbreviations

IBS, irritable bowel syndrome; FMT, fecal microbiota transplantation; PGE2, prostaglandin E2; TLR, Toll-like receptor; DC-SIGN, DC-specific ICAM3-grabbing non-integrin; NLRP3, NOD-like receptor protein 3; TJ, tight junction; TRPV1, transient reporter potential channel V1; PI-IBS, postinfectious-IBS; SARS-CoV-2, severe acute respiratory syndrome coronavirus-2; HCV, hepatitis C virus; RIG-1, retinoic acid inducible gene-1; MDA5, melanoma differentiation-associated gene 5; LGP2, laboratory of genetics and physiology 2; IL, interleukins; HLA, human leukocyte antigen; Th cell, T helper cell; DC, dendritic cell.

## References

[1] Mearin F., Lacy B.E., Chang L., Chey W.D., Lembo A.J., Simren M., Spiller R. (2016). Bowel Disorders. Gastroenterology.

[2] Sperber A.D., Dumitrascu D., Fukudo S., Gerson C., Ghoshal U.C., Gwee K.A., Hungin A.P.S., Kang J.Y., Minhu C., Schmulson M. (2017). The global prevalence of IBS in adults remains elusive due to the heterogeneity of studies: A Rome Foundation working team literature review. Gut.

[3] Hong G., Li Y., Yang M., Li G., Qian W., Xiong H., Bai T., Song J., Zhang L., Hou X. (2020). Gut fungal dysbiosis and altered bacterial-fungal interaction in patients with diarrhea-predominant irritable bowel syndrome: An explorative study. Neurogastroenterol. Motil..

[4] Vasant D.H., Paine P.A., Black C.J., Houghton L.A., Everitt H.A., Corsetti M., Agrawal A., Aziz I., Farmer A.D., Eugenicos M.P. (2021). British Society of Gastroenterology guidelines on the management of irritable bowel syndrome. Gut.

[5] Collins J., Borojevic R., Verdu E.F., Huizinga J.D., Ratcliffe E.M. (2014). Intestinal microbiota influence the early postnatal development of the enteric nervous system. Neurogastroenterol. Motil..

[6] Jeffery I.B., O’Toole P.W., Ohman L., Claesson M.J., Deane J., Quigley E.M., Simren M. (2012). An irritable bowel syndrome subtype defined by species-specific alterations in faecal microbiota. Gut.

[7] Rajilic-Stojanovic M., Biagi E., Heilig H.G., Kajander K., Kekkonen R.A., Tims S., de Vos W.M. (2011). Global and deep molecular analysis of microbiota signatures in fecal samples from patients with irritable bowel syndrome. Gastroenterology.

[8] Shukla R., Ghoshal U., Dhole T.N., Ghoshal U.C. (2015). Fecal Microbiota in Patients with Irritable Bowel Syndrome Compared with Healthy Controls Using Real-Time Polymerase Chain Reaction: An Evidence of Dysbiosis. Dig. Dis. Sci..

[9] Tap J., Derrien M., Tornblom H., Brazeilles R., Cools-Portier S., Dore J., Storsrud S., Le Neve B., Ohman L., Simren M. (2017). Identification of an Intestinal Microbiota Signature Associated With Severity of Irritable Bowel Syndrome. Gastroenterology.

[10] Macfarlane S., Woodmansey E.J., Macfarlane G.T. (2005). Colonization of mucin by human intestinal bacteria and establishment of biofilm communities in a two-stage continuous culture system. Appl. Environ. Microbiol..

[11] Pittayanon R., Lau J.T., Yuan Y., Leontiadis G.I., Tse F., Surette M., Moayyedi P. (2019). Gut Microbiota in Patients With Irritable Bowel Syndrome-A Systematic Review. Gastroenterology.

[12] O’Mahony L., McCarthy J., Kelly P., Hurley G., Luo F., Chen K., O’Sullivan G.C., Kiely B., Collins J.K., Shanahan F. (2005). Lactobacillus and bifidobacterium in irritable bowel syndrome: Symptom responses and relationship to cytokine profiles. Gastroenterology.

[13] Whorwell P.J., Altringer L., Morel J., Bond Y., Charbonneau D., O’Mahony L., Kiely B., Shanahan F., Quigley E.M. (2006). Efficacy of an encapsulated probiotic Bifidobacterium infantis 35624 in women with irritable bowel syndrome. Am. J. Gastroenterol..

[14] Gu Y., Zhou G., Qin X., Huang S., Wang B., Cao H. (2019). The Potential Role of Gut Mycobiome in Irritable Bowel Syndrome. Front. Microbiol..

[15] Pozuelo M., Panda S., Santiago A., Mendez S., Accarino A., Santos J., Guarner F., Azpiroz F., Manichanh C. (2015). Reduction of butyrate- and methane-producing microorganisms in patients with Irritable Bowel Syndrome. Sci. Rep..

[16] Botschuijver S., Roeselers G., Levin E., Jonkers D.M., Welting O., Heinsbroek S.E.M., de Weerd H.H., Boekhout T., Fornai M., Masclee A.A. (2017). Intestinal Fungal Dysbiosis Is Associated With Visceral Hypersensitivity in Patients With Irritable Bowel Syndrome and Rats. Gastroenterology.

[17] Ansari M.H., Ebrahimi M., Fattahi M.R., Gardner M.G., Safarpour A.R., Faghihi M.A., Lankarani K.B. (2020). Viral metagenomic analysis of fecal samples reveals an enteric virome signature in irritable bowel syndrome. BMC Microbiol..

[18] Qin J., Li R., Raes J., Arumugam M., Burgdorf K.S., Manichanh C., Nielsen T., Pons N., Levenez F., Yamada T. (2010). A human gut microbial gene catalogue established by metagenomic sequencing. Nature.

[19] Limon J.J., Skalski J.H., Underhill D.M. (2017). Commensal Fungi in Health and Disease. Cell Host Microbe.

[20] Das A., O’Herlihy E., Shanahan F., O’Toole P.W., Jeffery I.B. (2021). The fecal mycobiome in patients with Irritable Bowel Syndrome. Sci. Rep..

[21] Sciavilla P., Strati F., Di Paola M., Modesto M., Vitali F., Cavalieri D., Prati G.M., Di Vito M., Aragona G., De Filippo C. (2021). Gut microbiota profiles and characterization of cultivable fungal isolates in IBS patients. Appl. Microbiol. Biotechnol..

[22] Li Y., Hong G., Yang M., Li G., Jin Y., Xiong H., Qian W., Hou X. (2020). Fecal bacteria can predict the efficacy of rifaximin in patients with diarrhea-predominant irritable bowel syndrome. Pharmacol. Res..

[23] Ligaarden S.C., Lydersen S., Farup P.G. (2012). IgG and IgG4 antibodies in subjects with irritable bowel syndrome: A case control study in the general population. BMC Gastroenterol..

[24] Botschuijver S., Welting O., Levin E., Maria-Ferreira D., Koch E., Montijn R.C., Seppen J., Hakvoort T.B.M., Schuren F.H.J., de Jonge W.J. (2018). Reversal of visceral hypersensitivity in rat by Menthacarin((R)), a proprietary combination of essential oils from peppermint and caraway, coincides with mycobiome modulation. Neurogastroenterol. Motil..

[25] van Thiel I.A.M., Stavrou A.A., de Jong A., Theelen B., Davids M., Hakvoort T.B.M., Admiraal-van den Berg I., Weert I.C.M., de Kruijs M., Vu D. (2022). Genetic and phenotypic diversity of fecal Candida albicans strains in irritable bowel syndrome. Sci. Rep..

[26] Pinheiro I., Robinson L., Verhelst A., Marzorati M., Winkens B., den Abbeele P.V., Possemiers S. (2017). A yeast fermentate improves gastrointestinal discomfort and constipation by modulation of the gut microbiome: Results from a randomized double-blind placebo-controlled pilot trial. BMC Complement. Altern. Med..

[27] Gayathri R., Aruna T., Malar S., Shilpa B., Dhanasekar K.R. (2020). Efficacy of Saccharomyces cerevisiae CNCM I-3856 as an add-on therapy for irritable bowel syndrome. Int. J. Colorectal Dis..

[28] Constante M., De Palma G., Lu J., Jury J., Rondeau L., Caminero A., Collins S.M., Verdu E.F., Bercik P. (2021). Saccharomyces boulardii CNCM I-745 modulates the microbiota-gut-brain axis in a humanized mouse model of Irritable Bowel Syndrome. Neurogastroenterol. Motil..

[29] Santelmann H., Howard J.M. (2005). Yeast metabolic products, yeast antigens and yeasts as possible triggers for irritable bowel syndrome. Eur. J. Gastroenterol. Hepatol..

[30] Nahas R. (2011). Irritable bowel syndrome: Common integrative medicine perspectives. Chin. J. Integr. Med..

[31] Abbas Z., Yakoob J., Jafri W., Ahmad Z., Azam Z., Usman M.W., Shamim S., Islam M. (2014). Cytokine and clinical response to Saccharomyces boulardii therapy in diarrhea-dominant irritable bowel syndrome: A randomized trial. Eur. J. Gastroenterol. Hepatol..

[32] Spiller R., Pelerin F., Cayzeele Decherf A., Maudet C., Housez B., Cazaubiel M., Justen P. (2016). Randomized double blind placebo-controlled trial of Saccharomyces cerevisiae CNCM I-3856 in irritable bowel syndrome: Improvement in abdominal pain and bloating in those with predominant constipation. United Eur. Gastroenterol. J..

[33] Jawhara S., Mogensen E., Maggiotto F., Fradin C., Sarazin A., Dubuquoy L., Maes E., Guerardel Y., Janbon G., Poulain D. (2012). Murine model of dextran sulfate sodium-induced colitis reveals Candida glabrata virulence and contribution of beta-mannosyltransferases. J. Biol. Chem..

[34] Rizzetto L., Ifrim D.C., Moretti S., Tocci N., Cheng S.C., Quintin J., Renga G., Oikonomou V., De Filippo C., Weil T. (2016). Fungal Chitin Induces Trained Immunity in Human Monocytes during Cross-talk of the Host with Saccharomyces cerevisiae. J. Biol. Chem..

[35] Chiaro T.R., Soto R., Zac Stephens W., Kubinak J.L., Petersen C., Gogokhia L., Bell R., Delgado J.C., Cox J., Voth W. (2017). A member of the gut mycobiota modulates host purine metabolism exacerbating colitis in mice. Sci. Transl. Med..

[36] Bozza S., Fallarino F., Pitzurra L., Zelante T., Montagnoli C., Bellocchio S., Mosci P., Vacca C., Puccetti P., Romani L. (2005). A crucial role for tryptophan catabolism at the host/Candida albicans interface. J. Immunol..

[37] Polese B., Thurairajah B., Zhang H., Soo C.L., McMahon C.A., Fontes G., Hussain S.N.A., Abadie V., King I.L. (2021). Prostaglandin E2 amplifies IL-17 production by gammadelta T cells during barrier inflammation. Cell Rep..

[38] Grabauskas G., Wu X., Gao J., Li J.Y., Turgeon D.K., Owyang C. (2020). Prostaglandin E2, Produced by Mast Cells in Colon Tissues From Patients With Irritable Bowel Syndrome, Contributes to Visceral Hypersensitivity in Mice. Gastroenterology.

[39] Guilarte M., Santos J., de Torres I., Alonso C., Vicario M., Ramos L., Martinez C., Casellas F., Saperas E., Malagelada J.R. (2007). Diarrhoea-predominant IBS patients show mast cell activation and hyperplasia in the jejunum. Gut.

[40] Cremon C., Gargano L., Morselli-Labate A.M., Santini D., Cogliandro R.F., De Giorgio R., Stanghellini V., Corinaldesi R., Barbara G. (2009). Mucosal immune activation in irritable bowel syndrome: Gender-dependence and association with digestive symptoms. Am. J. Gastroenterol..

[41] Barbara G., Stanghellini V., De Giorgio R., Cremon C., Cottrell G.S., Santini D., Pasquinelli G., Morselli-Labate A.M., Grady E.F., Bunnett N.W. (2004). Activated mast cells in proximity to colonic nerves correlate with abdominal pain in irritable bowel syndrome. Gastroenterology.

[42] Ohman L., Simren M. (2010). Pathogenesis of IBS: Role of inflammation, immunity and neuroimmune interactions. Nat. Rev. Gastroenterol. Hepatol..

[43] Ohman L., Isaksson S., Lundgren A., Simren M., Sjovall H. (2005). A controlled study of colonic immune activity and beta7+ blood T lymphocytes in patients with irritable bowel syndrome. Clin. Gastroenterol. Hepatol..

[44] Gwee K.A., Collins S.M., Read N.W., Rajnakova A., Deng Y., Graham J.C., McKendrick M.W., Moochhala S.M. (2003). Increased rectal mucosal expression of interleukin 1beta in recently acquired post-infectious irritable bowel syndrome. Gut.

[45] Bennet S.M., Polster A., Tornblom H., Isaksson S., Capronnier S., Tessier A., Le Neve B., Simren M., Ohman L. (2016). Global Cytokine Profiles and Association With Clinical Characteristics in Patients With Irritable Bowel Syndrome. Am. J. Gastroenterol..

[46] Bashashati M., Rezaei N., Shafieyoun A., McKernan D.P., Chang L., Ohman L., Quigley E.M., Schmulson M., Sharkey K.A., Simren M. (2014). Cytokine imbalance in irritable bowel syndrome: A systematic review and meta-analysis. Neurogastroenterol. Motil..

[47] Netea M.G., Brown G.D., Kullberg B.J., Gow N.A. (2008). An integrated model of the recognition of Candida albicans by the innate immune system. Nat. Rev. Microbiol..

[48] Inoue M., Shinohara M.L. (2014). Clustering of pattern recognition receptors for fungal detection. PLoS Pathog..

[49] Bourgeois C., Kuchler K. (2012). Fungal pathogens-a sweet and sour treat for toll-like receptors. Front. Cell. Infect. Microbiol..

[50] Taylor P.R., Tsoni S.V., Willment J.A., Dennehy K.M., Rosas M., Findon H., Haynes K., Steele C., Botto M., Gordon S. (2007). Dectin-1 is required for beta-glucan recognition and control of fungal infection. Nat. Immunol..

[51] Koppel E.A., van Gisbergen K.P., Geijtenbeek T.B., van Kooyk Y. (2005). Distinct functions of DC-SIGN and its homologues L-SIGN (DC-SIGNR) and mSIGNR1 in pathogen recognition and immune regulation. Cell. Microbiol..

[52] Hise A.G., Tomalka J., Ganesan S., Patel K., Hall B.A., Brown G.D., Fitzgerald K.A. (2009). An essential role for the NLRP3 inflammasome in host defense against the human fungal pathogen Candida albicans. Cell Host Microbe.

[53] Lopes J.P., Stylianou M., Nilsson G., Urban C.F. (2015). Opportunistic pathogen Candida albicans elicits a temporal response in primary human mast cells. Sci. Rep..

[54] Roeder A., Kirschning C.J., Rupec R.A., Schaller M., Korting H.C. (2004). Toll-like receptors and innate antifungal responses. Trends Microbiol..

[55] LeibundGut-Landmann S., Gross O., Robinson M.J., Osorio F., Slack E.C., Tsoni S.V., Schweighoffer E., Tybulewicz V., Brown G.D., Ruland J. (2007). Syk- and CARD9-dependent coupling of innate immunity to the induction of T helper cells that produce interleukin 17. Nat. Immunol..

[56] Huppler A.R., Conti H.R., Hernandez-Santos N., Darville T., Biswas P.S., Gaffen S.L. (2014). Role of neutrophils in IL-17-dependent immunity to mucosal candidiasis. J. Immunol..

[57] Robinson M.J., Osorio F., Rosas M., Freitas R.P., Schweighoffer E., Gross O., Verbeek J.S., Ruland J., Tybulewicz V., Brown G.D. (2009). Dectin-2 is a Syk-coupled pattern recognition receptor crucial for Th17 responses to fungal infection. J. Exp. Med..

[58] Chen J., Zhang Y., Deng Z. (2012). Imbalanced shift of cytokine expression between T helper 1 and T helper 2 (Th1/Th2) in intestinal mucosa of patients with post-infectious irritable bowel syndrome. BMC Gastroenterol..

[59] Tan T.G., Lim Y.S., Tan A., Leong R., Pavelka N. (2019). Fungal Symbionts Produce Prostaglandin E2 to Promote Their Intestinal Colonization. Front. Cell. Infect. Microbiol..

[60] Rogiers O., Frising U.C., Kucharikova S., Jabra-Rizk M.A., van Loo G., Van Dijck P., Wullaert A. (2019). Candidalysin Crucially Contributes to Nlrp3 Inflammasome Activation by Candida albicans Hyphae. mBio.

[61] Kasper L., Konig A., Koenig P.A., Gresnigt M.S., Westman J., Drummond R.A., Lionakis M.S., Gross O., Ruland J., Naglik J.R. (2018). The fungal peptide toxin Candidalysin activates the NLRP3 inflammasome and causes cytolysis in mononuclear phagocytes. Nat. Commun..

[62] Moyes D.L., Wilson D., Richardson J.P., Mogavero S., Tang S.X., Wernecke J., Hofs S., Gratacap R.L., Robbins J., Runglall M. (2016). Candidalysin is a fungal peptide toxin critical for mucosal infection. Nature.

[63] Cheng S.C., van de Veerdonk F., Smeekens S., Joosten L.A., van der Meer J.W., Kullberg B.J., Netea M.G. (2010). Candida albicans dampens host defense by downregulating IL-17 production. J. Immunol..

[64] Marshall J.K., Thabane M., Garg A.X., Clark W., Meddings J., Collins S.M., Investigators W.E.L. (2004). Intestinal permeability in patients with irritable bowel syndrome after a waterborne outbreak of acute gastroenteritis in Walkerton, Ontario. Aliment. Pharmacol. Ther..

[65] Dunlop S.P., Hebden J., Campbell E., Naesdal J., Olbe L., Perkins A.C., Spiller R.C. (2006). Abnormal intestinal permeability in subgroups of diarrhea-predominant irritable bowel syndromes. Am. J. Gastroenterol..

[66] Piche T., Barbara G., Aubert P., Bruley des Varannes S., Dainese R., Nano J.L., Cremon C., Stanghellini V., De Giorgio R., Galmiche J.P. (2009). Impaired intestinal barrier integrity in the colon of patients with irritable bowel syndrome: Involvement of soluble mediators. Gut.

[67] Gecse K., Roka R., Ferrier L., Leveque M., Eutamene H., Cartier C., Ait-Belgnaoui A., Rosztoczy A., Izbeki F., Fioramonti J. (2008). Increased faecal serine protease activity in diarrhoeic IBS patients: A colonic lumenal factor impairing colonic permeability and sensitivity. Gut.

[68] Naglik J.R., Challacombe S.J., Hube B. (2003). Candida albicans secreted aspartyl proteinases in virulence and pathogenesis. Microbiol Mol. Biol. Rev..

[69] Phan Q.T., Myers C.L., Fu Y., Sheppard D.C., Yeaman M.R., Welch W.H., Ibrahim A.S., Edwards J.E., Filler S.G. (2007). Als3 is a Candida albicans invasin that binds to cadherins and induces endocytosis by host cells. PLoS Biol..

[70] Staab J.F., Bradway S.D., Fidel P.L., Sundstrom P. (1999). Adhesive and mammalian transglutaminase substrate properties of Candida albicans Hwp1. Science.

[71] Frank C.F., Hostetter M.K. (2007). Cleavage of E-cadherin: A mechanism for disruption of the intestinal epithelial barrier by Candida albicans. Transl. Res..

[72] Mao X., Qiu X., Jiao C., Lu M., Zhao X., Li X., Li J., Ma J., Zhang H. (2020). Candida albicans SC5314 inhibits NLRP3/NLRP6 inflammasome expression and dampens human intestinal barrier activity in Caco-2 cell monolayer model. Cytokine.

[73] Fidel P.L., Vazquez J.A., Sobel J.D. (1999). Candida glabrata: Review of epidemiology, pathogenesis, and clinical disease with comparison to C. albicans. Clin. Microbiol. Rev..

[74] Martinon F., Petrilli V., Mayor A., Tardivel A., Tschopp J. (2006). Gout-associated uric acid crystals activate the NALP3 inflammasome. Nature.

[75] Wang F., Graham W.V., Wang Y., Witkowski E.D., Schwarz B.T., Turner J.R. (2005). Interferon-gamma and tumor necrosis factor-alpha synergize to induce intestinal epithelial barrier dysfunction by up-regulating myosin light chain kinase expression. Am. J. Pathol..

[76] Bruewer M., Utech M., Ivanov A.I., Hopkins A.M., Parkos C.A., Nusrat A. (2005). Interferon-gamma induces internalization of epithelial tight junction proteins via a macropinocytosis-like process. FASEB J..

[77] Martinez C., Vicario M., Ramos L., Lobo B., Mosquera J.L., Alonso C., Sanchez A., Guilarte M., Antolin M., de Torres I. (2012). The jejunum of diarrhea-predominant irritable bowel syndrome shows molecular alterations in the tight junction signaling pathway that are associated with mucosal pathobiology and clinical manifestations. Am. J. Gastroenterol..

[78] Wouters M.M., Vicario M., Santos J. (2016). The role of mast cells in functional GI disorders. Gut.

[79] Annahazi A., Ferrier L., Bezirard V., Leveque M., Eutamene H., Ait-Belgnaoui A., Coeffier M., Ducrotte P., Roka R., Inczefi O. (2013). Luminal cysteine-proteases degrade colonic tight junction structure and are responsible for abdominal pain in constipation-predominant IBS. Am. J. Gastroenterol..

[80] Kelesidis T., Pothoulakis C. (2012). Efficacy and safety of the probiotic Saccharomyces boulardii for the prevention and therapy of gastrointestinal disorders. Ther. Adv. Gastroenterol..

[81] Jones R.C., Otsuka E., Wagstrom E., Jensen C.S., Price M.P., Gebhart G.F. (2007). Short-term sensitization of colon mechanoreceptors is associated with long-term hypersensitivity to colon distention in the mouse. Gastroenterology.

[82] Wouters M.M., Balemans D., Van Wanrooy S., Dooley J., Cibert-Goton V., Alpizar Y.A., Valdez-Morales E.E., Nasser Y., Van Veldhoven P.P., Vanbrabant W. (2016). Histamine Receptor H1-Mediated Sensitization of TRPV1 Mediates Visceral Hypersensitivity and Symptoms in Patients With Irritable Bowel Syndrome. Gastroenterology.

[83] Stanisor O.I., van Diest S.A., Yu Z., Welting O., Bekkali N., Shi J., de Jonge W.J., Boeckxstaens G.E., van den Wijngaard R.M. (2013). Stress-induced visceral hypersensitivity in maternally separated rats can be reversed by peripherally restricted histamine-1-receptor antagonists. PLoS ONE.

[84] Buhner S., Li Q., Vignali S., Barbara G., De Giorgio R., Stanghellini V., Cremon C., Zeller F., Langer R., Daniel H. (2009). Activation of human enteric neurons by supernatants of colonic biopsy specimens from patients with irritable bowel syndrome. Gastroenterology.

[85] Hughes P.A., Harrington A.M., Castro J., Liebregts T., Adam B., Grasby D.J., Isaacs N.J., Maldeniya L., Martin C.M., Persson J. (2013). Sensory neuro-immune interactions differ between irritable bowel syndrome subtypes. Gut.

[86] Zhou Q., Zhang B., Verne G.N. (2009). Intestinal membrane permeability and hypersensitivity in the irritable bowel syndrome. Pain.

[87] Martinez C., Lobo B., Pigrau M., Ramos L., Gonzalez-Castro A.M., Alonso C., Guilarte M., Guila M., de Torres I., Azpiroz F. (2013). Diarrhoea-predominant irritable bowel syndrome: An organic disorder with structural abnormalities in the jejunal epithelial barrier. Gut.

[88] Sovran B., Planchais J., Jegou S., Straube M., Lamas B., Natividad J.M., Agus A., Dupraz L., Glodt J., Da Costa G. (2018). Enterobacteriaceae are essential for the modulation of colitis severity by fungi. Microbiome.

[89] Garcia C., Tebbji F., Daigneault M., Liu N.N., Kohler J.R., Allen-Vercoe E., Sellam A. (2017). The Human Gut Microbial Metabolome Modulates Fungal Growth via the TOR Signaling Pathway. mSphere.

[90] Agus A., Planchais J., Sokol H. (2018). Gut Microbiota Regulation of Tryptophan Metabolism in Health and Disease. Cell Host Microbe.

[91] Zelante T., Iannitti R.G., Cunha C., De Luca A., Giovannini G., Pieraccini G., Zecchi R., D’Angelo C., Massi-Benedetti C., Fallarino F. (2013). Tryptophan catabolites from microbiota engage aryl hydrocarbon receptor and balance mucosal reactivity via interleukin-22. Immunity.

[92] Allonsius C.N., van den Broek M.F.L., De Boeck I., Kiekens S., Oerlemans E.F.M., Kiekens F., Foubert K., Vandenheuvel D., Cos P., Delputte P. (2017). Interplay between Lactobacillus rhamnosus GG and Candida and the involvement of exopolysaccharides. Microb. Biotechnol..

[93] Noverr M.C., Huffnagle G.B. (2004). Regulation of Candida albicans morphogenesis by fatty acid metabolites. Infect. Immun..

[94] Bhaskaran N., Quigley C., Paw C., Butala S., Schneider E., Pandiyan P. (2018). Role of Short Chain Fatty Acids in Controlling Tregs and Immunopathology During Mucosal Infection. Front. Microbiol..

[95] Allonsius C.N., Vandenheuvel D., Oerlemans E.F.M., Petrova M.I., Donders G.G.G., Cos P., Delputte P., Lebeer S. (2019). Inhibition of Candida albicans morphogenesis by chitinase from Lactobacillus rhamnosus GG. Sci. Rep..

[96] He L., Long C., Liu Y., Guo Y., Xiao N., Tan Z. (2017). Effects of Debaryomyces hansenii treatment on intestinal microorganisms in mice with antibiotics-induced diarrhea. 3 Biotech.

[97] Zeise K.D., Woods R.J., Huffnagle G.B. (2021). Interplay between Candida albicans and Lactic Acid Bacteria in the Gastrointestinal Tract: Impact on Colonization Resistance, Microbial Carriage, Opportunistic Infection, and Host Immunity. Clin. Microbiol. Rev..

[98] Botschuijver S., van Diest S.A., van Thiel I.A.M., Saia R.S., Strik A.S., Yu Z., Maria-Ferreira D., Welting O., Keszthelyi D., Jennings G. (2019). Miltefosine treatment reduces visceral hypersensitivity in a rat model for irritable bowel syndrome via multiple mechanisms. Sci. Rep..

[99] Biswas C., Zuo X., Chen S.C., Schibeci S.D., Forwood J.K., Jolliffe K.A., Sorrell T.C., Djordjevic J.T. (2014). Functional disruption of yeast metacaspase, Mca1, leads to miltefosine resistance and inability to mediate miltefosine-induced apoptotic effects. Fungal Genet. Biol..

[100] Widmer F., Wright L.C., Obando D., Handke R., Ganendren R., Ellis D.H., Sorrell T.C. (2006). Hexadecylphosphocholine (miltefosine) has broad-spectrum fungicidal activity and is efficacious in a mouse model of cryptococcosis. Antimicrob. Agents Chemother..

[101] Schulze J., Sonnenborn U. (2009). Yeasts in the gut: From commensals to infectious agents. Dtsch. Arztebl. Int..

[102] Ost K.S., O’Meara T.R., Stephens W.Z., Chiaro T., Zhou H., Penman J., Bell R., Catanzaro J.R., Song D., Singh S. (2021). Adaptive immunity induces mutualism between commensal eukaryotes. Nature.

[103] Ruben S., Garbe E., Mogavero S., Albrecht-Eckardt D., Hellwig D., Hader A., Kruger T., Gerth K., Jacobsen I.D., Elshafee O. (2020). Ahr1 and Tup1 Contribute to the Transcriptional Control of Virulence-Associated Genes in Candida albicans. mBio.

[104] Bockmuhl D.P., Krishnamurthy S., Gerads M., Sonneborn A., Ernst J.F. (2001). Distinct and redundant roles of the two protein kinase A isoforms Tpk1p and Tpk2p in morphogenesis and growth of Candida albicans. Mol. Microbiol..

[105] Lo H.J., Kohler J.R., DiDomenico B., Loebenberg D., Cacciapuoti A., Fink G.R. (1997). Nonfilamentous C. albicans mutants are avirulent. Cell.

[106] Mallick E.M., Bennett R.J. (2013). Sensing of the microbial neighborhood by Candida albicans. PLoS Pathog..

[107] Ramage G., Saville S.P., Wickes B.L., Lopez-Ribot J.L. (2002). Inhibition of Candida albicans biofilm formation by farnesol, a quorum-sensing molecule. Appl. Environ. Microbiol..

[108] Yan Y., Tan F., Miao H., Wang H., Cao Y. (2019). Effect of Shikonin Against Candida albicans Biofilms. Front. Microbiol..

[109] Murzyn A., Krasowska A., Stefanowicz P., Dziadkowiec D., Lukaszewicz M. (2010). Capric acid secreted by S. boulardii inhibits C. albicans filamentous growth, adhesion and biofilm formation. PLoS ONE.

[110] Terciolo C., Dobric A., Ouaissi M., Siret C., Breuzard G., Silvy F., Marchiori B., Germain S., Bonier R., Hama A. (2017). Saccharomyces boulardii CNCM I-745 Restores intestinal Barrier Integrity by Regulation of E-cadherin Recycling. J. Crohns Colitis.

[111] Cayzeele-Decherf A., Pelerin F., Leuillet S., Douillard B., Housez B., Cazaubiel M., Jacobson G.K., Justen P., Desreumaux P. (2017). Saccharomyces cerevisiae CNCM I-3856 in irritable bowel syndrome: An individual subject meta-analysis. World J. Gastroenterol..

[112] Pineton de Chambrun G., Neut C., Chau A., Cazaubiel M., Pelerin F., Justen P., Desreumaux P. (2015). A randomized clinical trial of Saccharomyces cerevisiae versus placebo in the irritable bowel syndrome. Dig. Liver Dis..

[113] Fraberger V., Call L.M., Domig K.J., D’Amico S. (2018). Applicability of Yeast Fermentation to Reduce Fructans and other FODMAPs. Nutrients.

[114] Struyf N., Laurent J., Verspreet J., Verstrepen K.J., Courtin C.M. (2017). Saccharomyces cerevisiae and Kluyveromyces marxianus Cocultures Allow Reduction of Fermentable Oligo-, Di-, and Monosaccharides and Polyols Levels in Whole Wheat Bread. J Agric. Food Chem..

[115] Li M., Luo T., Huang Y., Su J., Li D., Chen X., Zhang Y., Huang L., Li S., Jiao C. (2020). Polysaccharide from Pycnoporus sanguineus ameliorates dextran sulfate sodium-induced colitis via helper T cells repertoire modulation and autophagy suppression. Phytother. Res..

[116] Zhao D., Dai W., Tao H., Zhuang W., Qu M., Chang Y.N. (2020). Polysaccharide isolated from Auricularia auricular-judae (Bull.) prevents dextran sulfate sodium-induced colitis in mice through modulating the composition of the gut microbiota. J. Food Sci..

[117] Spagnuolo R., Cosco C., Mancina R.M., Ruggiero G., Garieri P., Cosco V., Doldo P. (2017). Beta-glucan, inositol and digestive enzymes improve quality of life of patients with inflammatory bowel disease and irritable bowel syndrome. Eur. Rev. Med. Pharmacol. Sci..

[118] Ciacci C., Franceschi F., Purchiaroni F., Capone P., Buccelletti F., Iacomini P., Ranaudo A., Andreozzi P., Tondi P., Gentiloni Silveri N. (2011). Effect of beta-glucan, inositol and digestive enzymes in GI symptoms of patients with IBS. Eur. Rev. Med. Pharmacol. Sci..

[119] Asano T., Tanaka K., Suemasu S., Ishihara T., Tahara K., Suzuki T., Suzuki H., Fukudo S., Mizushima T. (2012). Effects of beta-(1,3-1,6)-D-glucan on irritable bowel syndrome-related colonic hypersensitivity. Biochem. Biophys. Res. Commun..

[120] Johnsen P.H., Hilpusch F., Cavanagh J.P., Leikanger I.S., Kolstad C., Valle P.C., Goll R. (2018). Faecal microbiota transplantation versus placebo for moderate-to-severe irritable bowel syndrome: A double-blind, randomised, placebo-controlled, parallel-group, single-centre trial. Lancet Gastroenterol. Hepatol..

[121] Holvoet T., Joossens M., Wang J., Boelens J., Verhasselt B., Laukens D., van Vlierberghe H., Hindryckx P., De Vos M., De Looze D. (2017). Assessment of faecal microbial transfer in irritable bowel syndrome with severe bloating. Gut.

[122] Halkjaer S.I., Christensen A.H., Lo B.Z.S., Browne P.D., Gunther S., Hansen L.H., Petersen A.M. (2018). Faecal microbiota transplantation alters gut microbiota in patients with irritable bowel syndrome: Results from a randomised, double-blind placebo-controlled study. Gut.

[123] Zuo T., Wong S.H., Cheung C.P., Lam K., Lui R., Cheung K., Zhang F., Tang W., Ching J.Y.L., Wu J.C.Y. (2018). Gut fungal dysbiosis correlates with reduced efficacy of fecal microbiota transplantation in Clostridium difficile infection. Nat. Commun..

[124] Santiago-Rodriguez T.M., Hollister E.B. (2019). Human Virome and Disease: High-Throughput Sequencing for Virus Discovery, Identification of Phage-Bacteria Dysbiosis and Development of Therapeutic Approaches with Emphasis on the Human Gut. Viruses.

[125] Shkoporov A.N., Clooney A.G., Sutton T.D.S., Ryan F.J., Daly K.M., Nolan J.A., McDonnell S.A., Khokhlova E.V., Draper L.A., Forde A. (2019). The Human Gut Virome Is Highly Diverse, Stable, and Individual Specific. Cell Host Microbe.

[126] Gregory A.C., Zablocki O., Zayed A.A., Howell A., Bolduc B., Sullivan M.B. (2020). The Gut Virome Database Reveals Age-Dependent Patterns of Virome Diversity in the Human Gut. Cell Host Microbe.

[127] Aggarwala V., Liang G., Bushman F.D. (2017). Viral communities of the human gut: Metagenomic analysis of composition and dynamics. Mob. DNA.

[128] Reyes A., Haynes M., Hanson N., Angly F.E., Heath A.C., Rohwer F., Gordon J.I. (2010). Viruses in the faecal microbiota of monozygotic twins and their mothers. Nature.

[129] Norman J.M., Handley S.A., Baldridge M.T., Droit L., Liu C.Y., Keller B.C., Kambal A., Monaco C.L., Zhao G., Fleshner P. (2015). Disease-specific alterations in the enteric virome in inflammatory bowel disease. Cell.

[130] Zuo T., Lu X.J., Zhang Y., Cheung C.P., Lam S., Zhang F., Tang W., Ching J.Y.L., Zhao R., Chan P.K.S. (2019). Gut mucosal virome alterations in ulcerative colitis. Gut.

[131] Coughlan S., Das A., O’Herlihy E., Shanahan F., O’Toole P.W., Jeffery I.B. (2021). The gut virome in Irritable Bowel Syndrome differs from that of controls. Gut Microbes.

[132] Mihindukulasuriya K.A., Mars R.A.T., Johnson A.J., Ward T., Priya S., Lekatz H.R., Kalari K.R., Droit L., Zheng T., Blekhman R. (2021). Multi-Omics Analyses Show Disease, Diet, and Transcriptome Interactions With the Virome. Gastroenterology.

[133] Zanini B., Ricci C., Bandera F., Caselani F., Magni A., Laronga A.M., Lanzini A., San Felice del Benaco Study I. (2012). Incidence of post-infectious irritable bowel syndrome and functional intestinal disorders following a water-borne viral gastroenteritis outbreak. Am. J. Gastroenterol..

[134] Marshall J.K., Thabane M., Borgaonkar M.R., James C. (2007). Postinfectious irritable bowel syndrome after a food-borne outbreak of acute gastroenteritis attributed to a viral pathogen. Clin. Gastroenterol. Hepatol..

[135] Atmar R.L., Opekun A.R., Gilger M.A., Estes M.K., Crawford S.E., Neill F.H., Graham D.Y. (2008). Norwalk virus shedding after experimental human infection. Emerg. Infect. Dis..

[136] Buret A.G., Bhargava A. (2014). Modulatory mechanisms of enterocyte apoptosis by viral, bacterial and parasitic pathogens. Crit. Rev. Microbiol..

[137] Spiller R., Garsed K. (2009). Infection, inflammation, and the irritable bowel syndrome. Dig. Liver Dis..

[138] Carfi A., Bernabei R., Landi F., Gemelli Against C.-P.-A.C.S.G. (2020). Persistent Symptoms in Patients After Acute COVID-19. JAMA.

[139] Ghoshal U.C., Ghoshal U., Rahman M.M., Mathur A., Rai S., Akhter M., Mostafa T., Islam M.S., Haque S.A., Pandey A. (2022). Post-infection functional gastrointestinal disorders following coronavirus disease-19: A case-control study. J. Gastroenterol. Hepatol.

[140] Britton G.J., Chen-Liaw A., Cossarini F., Livanos A.E., Spindler M.P., Plitt T., Eggers J., Mogno I., Gonzalez-Reiche A.S., Siu S. (2021). Limited intestinal inflammation despite diarrhea, fecal viral RNA and SARS-CoV-2-specific IgA in patients with acute COVID-19. Sci. Rep..

[141] Jiang X., Luo M., Zou Z., Wang X., Chen C., Qiu J. (2020). Asymptomatic SARS-CoV-2 infected case with viral detection positive in stool but negative in nasopharyngeal samples lasts for 42 days. J. Med. Virol..

[142] Lamers M.M., Beumer J., van der Vaart J., Knoops K., Puschhof J., Breugem T.I., Ravelli R.B.G., Paul van Schayck J., Mykytyn A.Z., Duimel H.Q. (2020). SARS-CoV-2 productively infects human gut enterocytes. Science.

[143] Hoffmann M., Kleine-Weber H., Schroeder S., Kruger N., Herrler T., Erichsen S., Schiergens T.S., Herrler G., Wu N.H., Nitsche A. (2020). SARS-CoV-2 Cell Entry Depends on ACE2 and TMPRSS2 and Is Blocked by a Clinically Proven Protease Inhibitor. Cell.

[144] Wang Q., Zhang Y., Wu L., Niu S., Song C., Zhang Z., Lu G., Qiao C., Hu Y., Yuen K.Y. (2020). Structural and Functional Basis of SARS-CoV-2 Entry by Using Human ACE2. Cell.

[145] Riley T.R., Chinchilli V.M., Shoemaker M., Koch K. (2001). Is nausea associated with chronic hepatitis C infection?. Am. J. Gastroenterol..

[146] Fontana R.J., Moyer C.A., Sonnad S., Lok A.S.F., Sneed-Pee N., Walsh J., Klein S., Webster S. (2001). Comorbidities and quality of life in patients with interferon-refractory chronic hepatitis C. Am. J. Gastroenterol..

[147] Fouad Y.M., Makhlouf M.M., Khalaf H., Mostafa Z., Abdel Raheem E., Meneasi W. (2010). Is irritable bowel syndrome associated with chronic hepatitis C?. J. Gastroenterol. Hepatol..

[148] Shimada T., Nagata N., Okahara K., Joya A., Hayashida T., Oka S., Sakurai T., Akiyama J., Uemura N., Gatanaga H. (2017). PCR detection of human herpesviruses in colonic mucosa of individuals with inflammatory bowel disease: Comparison with individuals with immunocompetency and HIV infection. PLoS ONE.

[149] Nellaker C., Yao Y., Jones-Brando L., Mallet F., Yolken R.H., Karlsson H. (2006). Transactivation of elements in the human endogenous retrovirus W family by viral infection. Retrovirology.

[150] Alexopoulou L., Holt A.C., Medzhitov R., Flavell R.A. (2001). Recognition of double-stranded RNA and activation of NF-kappaB by Toll-like receptor 3. Nature.

[151] Heil F., Hemmi H., Hochrein H., Ampenberger F., Kirschning C., Akira S., Lipford G., Wagner H., Bauer S. (2004). Species-specific recognition of single-stranded RNA via toll-like receptor 7 and 8. Science.

[152] Lund J., Sato A., Akira S., Medzhitov R., Iwasaki A. (2003). Toll-like receptor 9-mediated recognition of Herpes simplex virus-2 by plasmacytoid dendritic cells. J. Exp. Med..

[153] Schlee M., Roth A., Hornung V., Hagmann C.A., Wimmenauer V., Barchet W., Coch C., Janke M., Mihailovic A., Wardle G. (2009). Recognition of 5’ triphosphate by RIG-I helicase requires short blunt double-stranded RNA as contained in panhandle of negative-strand virus. Immunity.

[154] Wu B., Peisley A., Richards C., Yao H., Zeng X., Lin C., Chu F., Walz T., Hur S. (2013). Structural basis for dsRNA recognition, filament formation, and antiviral signal activation by MDA5. Cell.

[155] Bruns A.M., Leser G.P., Lamb R.A., Horvath C.M. (2014). The innate immune sensor LGP2 activates antiviral signaling by regulating MDA5-RNA interaction and filament assembly. Mol. Cell.

[156] Galtier M., De Sordi L., Sivignon A., de Vallee A., Maura D., Neut C., Rahmouni O., Wannerberger K., Darfeuille-Michaud A., Desreumaux P. (2017). Bacteriophages Targeting Adherent Invasive Escherichia coli Strains as a Promising New Treatment for Crohn’s Disease. J. Crohns Colitis.

[157] Murakami K., Habukawa C., Nobuta Y., Moriguchi N., Takemura T. (2012). The effect of Lactobacillus brevis KB290 against irritable bowel syndrome: A placebo-controlled double-blind crossover trial. Biopsychosoc. Med..

[158] Mihara T., Nishimura Y., Shimizu Y., Nishiyama H., Yoshikawa G., Uehara H., Hingamp P., Goto S., Ogata H. (2016). Linking Virus Genomes with Host Taxonomy. Viruses.

[159] Barton E.S., White D.W., Cathelyn J.S., Brett-McClellan K.A., Engle M., Diamond M.S., Miller V.L., Virgin H.W.t. (2007). Herpesvirus latency confers symbiotic protection from bacterial infection. Nature.

[160] McElrath C., Espinosa V., Lin J.D., Peng J., Sridhar R., Dutta O., Tseng H.C., Smirnov S.V., Risman H., Sandoval M.J. (2021). Critical role of interferons in gastrointestinal injury repair. Nat. Commun..

[161] Leibowitz B.J., Zhao G., Wei L., Ruan H., Epperly M., Chen L., Lu X., Greenberger J.S., Zhang L., Yu J. (2021). Interferon b drives intestinal regeneration after radiation. Sci. Adv..

[162] Lurie-Weinberger M.N., Gophna U. (2015). Archaea in and on the Human Body: Health Implications and Future Directions. PLoS Pathog..

[163] Moissl-Eichinger C., Probst A.J., Birarda G., Auerbach A., Koskinen K., Wolf P., Holman H.N. (2017). Human age and skin physiology shape diversity and abundance of Archaea on skin. Sci. Rep..

[164] Blais Lecours P., Marsolais D., Cormier Y., Berberi M., Hache C., Bourdages R., Duchaine C. (2014). Increased prevalence of Methanosphaera stadtmanae in inflammatory bowel diseases. PLoS ONE.

[165] Koskinen K., Pausan M.R., Perras A.K., Beck M., Bang C., Mora M., Schilhabel A., Schmitz R., Moissl-Eichinger C. (2017). First Insights into the Diverse Human Archaeome: Specific Detection of Archaea in the Gastrointestinal Tract, Lung, and Nose and on Skin. mBio.

[166] Walters W., Hyde E.R., Berg-Lyons D., Ackermann G., Humphrey G., Parada A., Gilbert J.A., Jansson J.K., Caporaso J.G., Fuhrman J.A. (2016). Improved Bacterial 16S rRNA Gene (V4 and V4-5) and Fungal Internal Transcribed Spacer Marker Gene Primers for Microbial Community Surveys. mSystems.

[167] Wampach L., Heintz-Buschart A., Hogan A., Muller E.E.L., Narayanasamy S., Laczny C.C., Hugerth L.W., Bindl L., Bottu J., Andersson A.F. (2017). Colonization and Succession within the Human Gut Microbiome by Archaea, Bacteria, and Microeukaryotes during the First Year of Life. Front. Microbiol..

[168] Attaluri A., Jackson M., Valestin J., Rao S.S. (2010). Methanogenic flora is associated with altered colonic transit but not stool characteristics in constipation without IBS. Am. J. Gastroenterol..

[169] Sahakian A.B., Jee S.R., Pimentel M. (2010). Methane and the gastrointestinal tract. Dig. Dis. Sci..

[170] Ghoshal U., Shukla R., Srivastava D., Ghoshal U.C. (2016). Irritable Bowel Syndrome, Particularly the Constipation-Predominant Form, Involves an Increase in Methanobrevibacter smithii, Which Is Associated with Higher Methane Production. Gut Liver.

[171] Kim G., Deepinder F., Morales W., Hwang L., Weitsman S., Chang C., Gunsalus R., Pimentel M. (2012). Methanobrevibacter smithii is the predominant methanogen in patients with constipation-predominant IBS and methane on breath. Dig. Dis. Sci..

[172] Chassard C., Dapoigny M., Scott K.P., Crouzet L., Del’homme C., Marquet P., Martin J.C., Pickering G., Ardid D., Eschalier A. (2012). Functional dysbiosis within the gut microbiota of patients with constipated-irritable bowel syndrome. Aliment Pharmacol. Ther..

[173] Rana S.V., Sharma S., Sinha S.K., Kaur H., Sikander A., Singh K. (2009). Incidence of predominant methanogenic flora in irritable bowel syndrome patients and apparently healthy controls from North India. Dig. Dis. Sci..

[174] Edwinson A., Yang L., Chen J., Grover M. (2021). Colonic expression of Ace2, the SARS-CoV-2 entry receptor, is suppressed by commensal human microbiota. Gut Microbes.

[175] Vierbuchen T., Bang C., Rosigkeit H., Schmitz R.A., Heine H. (2017). The Human-Associated Archaeon Methanosphaera stadtmanae Is Recognized through Its RNA and Induces TLR8-Dependent NLRP3 Inflammasome Activation. Front. Immunol..

[176] Bang C., Ehlers C., Orell A., Prasse D., Spinner M., Gorb S.N., Albers S.V., Schmitz R.A. (2014). Biofilm formation of mucosa-associated methanoarchaeal strains. Front. Microbiol..

[177] Samuel B.S., Hansen E.E., Manchester J.K., Coutinho P.M., Henrissat B., Fulton R., Latreille P., Kim K., Wilson R.K., Gordon J.I. (2007). Genomic and metabolic adaptations of Methanobrevibacter smithii to the human gut. Proc. Natl. Acad. Sci. USA.

[178] Samuel B.S., Gordon J.I. (2006). A humanized gnotobiotic mouse model of host-archaeal-bacterial mutualism. Proc. Natl. Acad. Sci. USA.

[179] Million M., Tidjani Alou M., Khelaifia S., Bachar D., Lagier J.C., Dione N., Brah S., Hugon P., Lombard V., Armougom F. (2016). Increased Gut Redox and Depletion of Anaerobic and Methanogenic Prokaryotes in Severe Acute Malnutrition. Sci. Rep..

[180] Gandhi A., Shah A., Jones M.P., Koloski N., Talley N.J., Morrison M., Holtmann G. (2021). Methane positive small intestinal bacterial overgrowth in inflammatory bowel disease and irritable bowel syndrome: A systematic review and meta-analysis. Gut Microbes.

[181] Pimentel M., Mayer A.G., Park S., Chow E.J., Hasan A., Kong Y. (2003). Methane production during lactulose breath test is associated with gastrointestinal disease presentation. Dig. Dis. Sci..

[182] Kunkel D., Basseri R.J., Makhani M.D., Chong K., Chang C., Pimentel M. (2011). Methane on breath testing is associated with constipation: A systematic review and meta-analysis. Dig. Dis. Sci..

[183] Hwang L., Low K., Khoshini R., Melmed G., Sahakian A., Makhani M., Pokkunuri V., Pimentel M. (2010). Evaluating breath methane as a diagnostic test for constipation-predominant IBS. Dig. Dis. Sci..

[184] Pimentel M., Lin H.C., Enayati P., van den Burg B., Lee H.R., Chen J.H., Park S., Kong Y., Conklin J. (2006). Methane, a gas produced by enteric bacteria, slows intestinal transit and augments small intestinal contractile activity. Am. J. Physiol. Gastrointest. Liver Physiol..

[185] Jahng J., Jung I.S., Choi E.J., Conklin J.L., Park H. (2012). The effects of methane and hydrogen gases produced by enteric bacteria on ileal motility and colonic transit time. Neurogastroenterol. Motil..

[186] Bond J.H., Engel R.R., Levitt M.D. (1971). Factors influencing pulmonary methane excretion in man. An indirect method of studying the in situ metabolism of the methane-producing colonic bacteria. J. Exp. Med..

[187] Rezaie A., Buresi M., Lembo A., Lin H., McCallum R., Rao S., Schmulson M., Valdovinos M., Zakko S., Pimentel M. (2017). Hydrogen and Methane-Based Breath Testing in Gastrointestinal Disorders: The North American Consensus. Am. J. Gastroenterol..

[188] Levitt M.D., Furne J.K., Kuskowski M., Ruddy J. (2006). Stability of human methanogenic flora over 35 years and a review of insights obtained from breath methane measurements. Clin. Gastroenterol. Hepatol..

[189] Pimentel M., Chang C., Chua K.S., Mirocha J., DiBaise J., Rao S., Amichai M. (2014). Antibiotic treatment of constipation-predominant irritable bowel syndrome. Dig. Dis. Sci..

[190] Gottlieb K., Wacher V., Sliman J., Pimentel M. (2016). Review article: Inhibition of methanogenic archaea by statins as a targeted management strategy for constipation and related disorders. Aliment. Pharmacol. Ther..

[191] Hubert S., Chadwick A., Wacher V., Coughlin O., Kokai-Kun J., Bristol A. (2018). Development of a Modified-Release Formulation of Lovastatin Targeted to Intestinal Methanogens Implicated in Irritable Bowel Syndrome With Constipation. J. Pharm. Sci..

[192] Miller T.L., Wolin M.J. (2001). Inhibition of growth of methane-producing bacteria of the ruminant forestomach by hydroxymethylglutaryl-SCoA reductase inhibitors. J. Dairy Sci..

[193] Muskal S.M., Sliman J., Kokai-Kun J., Pimentel M., Wacher V., Gottlieb K. (2016). Lovastatin lactone may improve irritable bowel syndrome with constipation (IBS-C) by inhibiting enzymes in the archaeal methanogenesis pathway. F1000Research.

[194] Katsnelson A. (2016). Diagnostics: Filling in the missing pieces. Nature.

[195] Saha L. (2014). Irritable bowel syndrome: Pathogenesis, diagnosis, treatment, and evidence-based medicine. World J. Gastroenterol..

[196] Abedi S.H., Fazlzadeh A., Mollalo A., Sartip B., Mahjour S., Bahadory S., Taghipour A., Rostami A. (2022). The neglected role of Blastocystis sp. and Giardia lamblia in development of irritable bowel syndrome: A systematic review and meta-analysis. Microb. Pathog..

[197] Nakao J.H., Collier S.A., Gargano J.W. (2017). Giardiasis and Subsequent Irritable Bowel Syndrome: A Longitudinal Cohort Study Using Health Insurance Data. J. Infect. Dis..

[198] Rehn M., Wallensten A., Widerstrom M., Lilja M., Grunewald M., Stenmark S., Kark M., Lindh J. (2015). Post-infection symptoms following two large waterborne outbreaks of Cryptosporidium hominis in Northern Sweden, 2010–2011. BMC Public Health.

[199] Carter B.L., Stiff R.E., Elwin K., Hutchings H.A., Mason B.W., Davies A.P., Chalmers R.M. (2019). Health sequelae of human cryptosporidiosis-a 12-month prospective follow-up study. Eur. J. Clin. Microbiol. Infect. Dis..

[200] Soyturk M., Akpinar H., Gurler O., Pozio E., Sari I., Akar S., Akarsu M., Birlik M., Onen F., Akkoc N. (2007). Irritable bowel syndrome in persons who acquired trichinellosis. Am. J. Gastroenterol..

[201] Krogsgaard L.R., Andersen L.O., Johannesen T.B., Engsbro A.L., Stensvold C.R., Nielsen H.V., Bytzer P. (2018). Characteristics of the bacterial microbiome in association with common intestinal parasites in irritable bowel syndrome. Clin. Transl. Gastroenterol..

[202] Krogsgaard L.R., Engsbro A.L., Stensvold C.R., Nielsen H.V., Bytzer P. (2015). The prevalence of intestinal parasites is not greater among individuals with irritable bowel syndrome: A population-based case-control study. Clin. Gastroenterol. Hepatol..

[203] Stark D., Beebe N., Marriott D., Ellis J., Harkness J. (2005). Prospective study of the prevalence, genotyping, and clinical relevance of Dientamoeba fragilis infections in an Australian population. J. Clin. Microbiol..

[204] Chen T.L., Chen S., Wu H.W., Lee T.C., Lu Y.Z., Wu L.L., Ni Y.H., Sun C.H., Yu W.H., Buret A.G. (2013). Persistent gut barrier damage and commensal bacterial influx following eradication of Giardia infection in mice. Gut Pathog..

[205] Fisher B.S., Estrano C.E., Cole J.A. (2013). Modeling long-term host cell-Giardia lamblia interactions in an in vitro co-culture system. PLoS ONE.

[206] Stadelmann B., Hanevik K., Andersson M.K., Bruserud O., Svard S.G. (2013). The role of arginine and arginine-metabolizing enzymes during Giardia-host cell interactions in vitro. BMC Microbiol..

[207] Dizdar V., Spiller R., Singh G., Hanevik K., Gilja O.H., El-Salhy M., Hausken T. (2010). Relative importance of abnormalities of CCK and 5-HT (serotonin) in Giardia-induced post-infectious irritable bowel syndrome and functional dyspepsia. Aliment. Pharmacol. Ther..

[208] Leslie F.C., Thompson D.G., McLaughlin J.T., Varro A., Dockray G.J., Mandal B.K. (2003). Plasma cholecystokinin concentrations are elevated in acute upper gastrointestinal infections. QJM.

[209] Chey W.Y., Jin H.O., Lee M.H., Sun S.W., Lee K.Y. (2001). Colonic motility abnormality in patients with irritable bowel syndrome exhibiting abdominal pain and diarrhea. Am. J. Gastroenterol..

[210] Zhang H., Yan Y., Shi R., Lin Z., Wang M., Lin L. (2008). Correlation of gut hormones with irritable bowel syndrome. Digestion.

[211] El-Gayar E.K., Mokhtar A.B., Hassan W.A. (2016). Study of the pathogenic potential of Dientamoeba fragilis in experimentally infected mice. Parasite Epidemiol. Control..

[212] Qin H.Y., Wu J.C., Tong X.D., Sung J.J., Xu H.X., Bian Z.X. (2011). Systematic review of animal models of post-infectious/post-inflammatory irritable bowel syndrome. J. Gastroenterol..

[213] Akiho H., Deng Y., Blennerhassett P., Kanbayashi H., Collins S.M. (2005). Mechanisms underlying the maintenance of muscle hypercontractility in a model of postinfective gut dysfunction. Gastroenterology.

[214] Keating C., Beyak M., Foley S., Singh G., Marsden C., Spiller R., Grundy D. (2008). Afferent hypersensitivity in a mouse model of post-inflammatory gut dysfunction: Role of altered serotonin metabolism. J. Physiol..

[215] Aerssens J., Hillsley K., Peeters P.J., de Hoogt R., Stanisz A., Lin J.H., Van den Wyngaert I., Gohlmann H.W., Grundy D., Stead R.H. (2007). Alterations in the brain-gut axis underlying visceral chemosensitivity in Nippostrongylus brasiliensis-infected mice. Gastroenterology.

[216] Khaldi S., Gargala G., Le Goff L., Parey S., Francois A., Fioramonti J., Ballet J.J., Dupont J.P., Ducrotte P., Favennec L. (2009). Cryptosporidium parvum isolate-dependent postinfectious jejunal hypersensitivity and mast cell accumulation in an immunocompetent rat model. Infect. Immun..

[217] Jawhara S., Habib K., Maggiotto F., Pignede G., Vandekerckove P., Maes E., Dubuquoy L., Fontaine T., Guerardel Y., Poulain D. (2012). Modulation of intestinal inflammation by yeasts and cell wall extracts: Strain dependence and unexpected anti-inflammatory role of glucan fractions. PLoS ONE.

